# Tumor segmentation via enhanced area growth algorithm for lung CT images

**DOI:** 10.1186/s12880-023-01126-y

**Published:** 2023-11-20

**Authors:** Abdollah Khorshidi

**Affiliations:** School of Paramedical, Gerash University of Medical Sciences, P.O. Box: 7441758666, Gerash, Iran

**Keywords:** Enhance area growth, Contrast augmentation, Start point, Automatic thresholding, Edge improvement, Tumor borders, Accuracy, Acceptance rate, Comparison quantity, Computed tomography, MATLAB software

## Abstract

**Background:**

Since lung tumors are in dynamic conditions, the study of tumor growth and its changes is of great importance in primary diagnosis.

**Methods:**

Enhanced area growth (EAG) algorithm is introduced to segment the lung tumor in 2D and 3D modes on 60 patients CT images from four different databases by MATLAB software. The contrast augmentation, color intensity and maximum primary tumor radius determination, thresholding, start and neighbor points’ designation in an array, and then modifying the points in the braid on average are the early steps of the proposed algorithm. To determine the new tumor boundaries, the maximum distance from the color-intensity center point of the primary tumor to the modified points is appointed via considering a larger target region and new threshold. The tumor center is divided into different subsections and then all previous stages are repeated from new designated points to define diverse boundaries for the tumor. An interpolation between these boundaries creates a new tumor boundary. The intersections with the tumor boundaries are firmed for edge correction phase, after drawing diverse lines from the tumor center at relevant angles. Each of the new regions is annexed to the core region to achieve a segmented tumor surface by meeting certain conditions.

**Results:**

The multipoint-growth-starting-point grouping fashioned a desired consequence in the precise delineation of the tumor. The proposed algorithm enhanced tumor identification by more than 16% with a reasonable accuracy acceptance rate. At the same time, it largely assurances the independence of the last outcome from the starting point. By significance difference of *p* < 0.05, the dice coefficients were 0.80 ± 0.02 and 0.92 ± 0.03, respectively, for primary and enhanced algorithms. Lung area determination alongside automatic thresholding and also starting from several points along with edge improvement may reduce human errors in radiologists’ interpretation of tumor areas and selection of the algorithm’s starting point.

**Conclusions:**

The proposed algorithm enhanced tumor detection by more than 18% with a sufficient acceptance ratio of accuracy. Since the enhanced algorithm is independent of matrix size and image thickness, it is very likely that it can be easily applied to other contiguous tumor images.

**Trial registration:**

PAZHOUHAN, PAZHOUHAN98000032. Registered 4 January 2021, http://pazhouhan.gerums.ac.ir/webreclist/view.action?webreclist_code=19300

## Introduction

Treatment using radiation is a helpful technique to compensate and supplement the lack of chemotherapy to curb and prevent tumor growth. The optimization takes into account the least harm to healthy tissue and the greatest possible harm to the tumor. First, the plan of the treatment is appraised to establish the field size, radiation angle and prescribed dose using size and position of the tumor. Lung tumors are one of the main varieties of tumors that lead to death in humans. The purpose of this research is to recognize the lung tumor using reported X-ray computed tomography (CT) images. Usually localization and subdivision are utilized as an influential instrument in processing of medical images. These utilizations include the detection of edges and tumor sites in the image, the identification of the tumor surface and then the postoperative diagnostic phase [1, 2].

Hu et al. 2001 [3] have represented an automatic method for segmentation the lungs images from 3D pulmonary X-ray CT imaging, in which the root mean square difference between the computer and human analysis was 0.8 pixels, 0.54 mm, as averaged over all volumes. Silva et al. 2001 [4] have combined adaptive intensity discrimination and geometrical feature in contour extraction and asserted that their algorithm demonstrates a greater agreement to any of radiologists than two radiologists between them. Also, Leader et al. 2003 [5] have evaluated 101 patients via a proposed lung segmentation including size, circularity and locations features by 95% accuracy and 95 ± 52 mL mean difference of the total lung volume. Armato et al. 2004 [6] used gray-level thresholding to segment the lungs and eliminate the trachea and main bronchi beside suppression of the diaphragm in pleural mesothelioma tumor assessment. Meanwhile, Pu et al. 2008 [7] have introduced a geometric algorithm to smooth the lung border considering juxtapleural nodules and adjacent regions by volumetric improvement during one minute. Moreover, Prasad et al. [8] have demonstrated a multi-threshold iterative method through polynomial interpolation and morphologic operations to resolve the curvature of the lung border line to that of the ribs. Simply, some researches have shown that the 20% neighboring is reasonable and convinced in thresholding. On the other hand, Pu et al. [9] have presented a technique with radial fitting via defining implicit function to eliminate troublesome areas and find an exclusive surface border through breaking besides repairing procedures. Rios Velazquez et al. [10] evaluated semiautomatic-segmented and manual volumes besides common fractions in macroscopic abnormal lung with restrictions in pathological tumor length. But determination of optimal surface via segmentation has been reviewed by Sun et al. [11] by means of a mixed virtual-desktop reality user interface.

The work presented here is part of a larger effort to develop semi-automatic organ segmentation methods that speed up and improve the accuracy of the chest and breast cancer treatment planning process [12–15]. In this way and through considering respiratory system, it is crucially important to accurately segment different organs such as lungs to facilitate the quantitative analysis and visualization of the clinically significant features toward the diagnosis, treatment planning and follow-up evaluation. Among several different segmentation methods, those that are deformation-based are especially appealing for our application because they can provide smooth boundary and accurately capture the high-curvature features of the lung regions of different patients. This is due to the ability of multi-point edge detection algorithm to segment anatomical structures with prior knowledge about the location, size, and shape of the structures. Amini et al. [16] have suggested a dynamic programming algorithm for minimizing function energy, which lets adding severe restraints to achieve an appropriate behavior of the particular images. However, the projected algorithm is slow and has a large complexity in number of points in the contour and also in size of the neighborhood a point that can exchange during a distinct iteration stage [17].

Meanwhile, Cohen [17] has suggested an additional force that caused the curve to perform like a balloon being inflated through this supplementary force. In addition to that, Williams and Shah [18] have developed a greedy algorithm whose performance is comparable to the dynamic programming and the method of calculus of variations. They have revealed dissimilar procedures for continuity term and appraised several estimates for defined curvature term. Relatively, it has been found to be comparable in final results, while having less computational cost than dynamic programming with lower complexity and being more flexible and stable for incorporating severe restraints than the calculus of variations method. On the other hand, Radeva et al. [19] have suggested another procedure that include the gradient orientation of the image edge points and implement an external force besides a novel potential field to organize deformation convergence and attraction by both far and near edges [20]. Park and Keller [21] introduced a new procedure merging watershed transformation and dynamic programming and called it Watersnake. This new snake procedure is normally utilized to choose which points are needed to eliminate unnecessary curves while preserving important curves.

Here by this research, an enhanced area growth (EAG) algorithm is simply defined to expedite the area growth in lung tumor segmentation with an enhanced accuracy. The obtained results are compared to manual segmentations of the lung provided by an expert radiologist and with those of previous works, showing encouraging results and high robustness of our approach.

## Methods

**Fig. 1:**
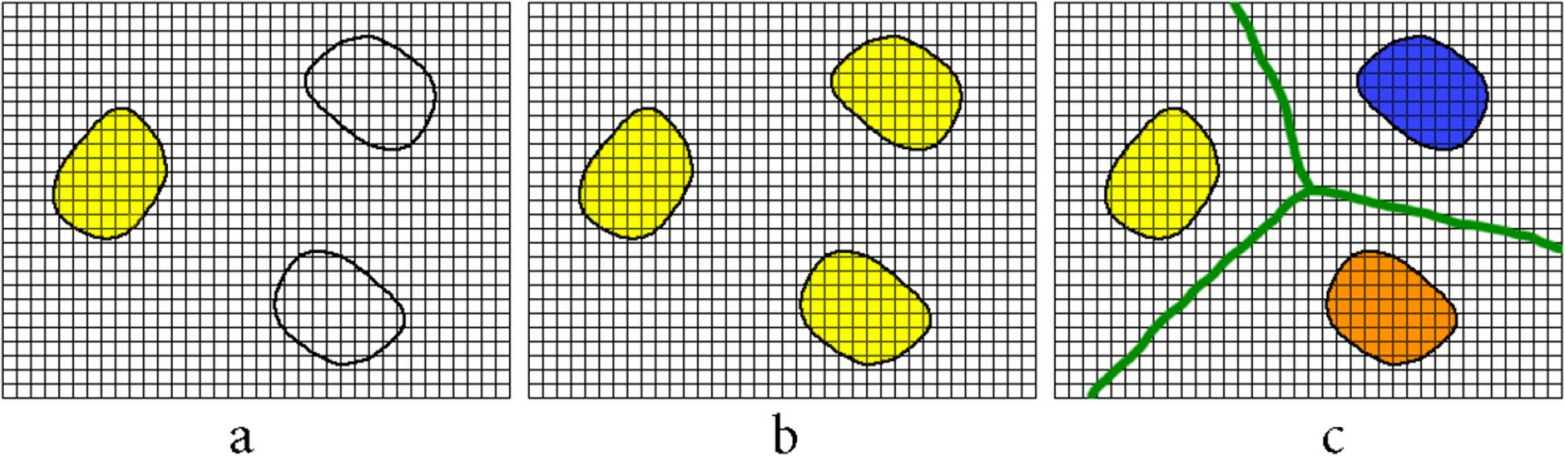
Types of region growing methods: **a**) Regional, **b**) Global, **c**) Splitting and merging

The area growth is one of the methods of segmentation and clustering in image processing. The basis of this method is to start from a point in the selected region and grow based on common features of the neighboring points and finally complete clustering of the segment. In general, this method is used in three ways: 1) Regional method: Segmentation is done only for points that are adjacent to each other without any discontinuity. In this method, the neighborhood is an essential condition for being in a segment. 2) Global method: The neighborhood of points is not a criterion and the existence of a common feature in all points of the image is examined. Then, all points that have common features, like color intensity of spots, are segmented. 3) Splitting and merging: This method is done in two phases. In the first phase, the image is subdivided into different zones and the regional algorithm is applied in each of the zones. Then in the second phase, the results of all the algorithms are merged. Figure 1 compares these three methods.

Since the lung tumor is usually concentrated in a specific area, therefore in this study, the regional approach is examined. In this approach, the growth is started from a point which is set by the user. It seems quite reasonable because the diagnosis of tumor position requires a thorough understanding of the lung anatomy and cannot be done fully automatically. First, the user requires determining a point of the tumor, and then all the steps are automatically performed to reach the limit of the desired region of interest (ROI) with a certain constraint and find the tumor edges in segmentation. The region growing is a method that can be implemented on both 2D and 3D images.

### Primary area growth algorithm for 2D and 3D images

The primary algorithm introduced for growing area in 2D images is as follows:

1) The desired 2D image of a lung including a tumor is selected and named an image.

2) After *lungarea* appointing, the user decides the starting-pixel coordinate for area growth.

3) The *seedval* command saves the primary value based on the color intensity of appointed pixel.

4) The *threshval* command assesses the threshold by 20% gray-level default on the whole image.

1 $$threshval=20\mathrm{\%*graythresh}\left(imag\right)$$

**Fig. 2:**
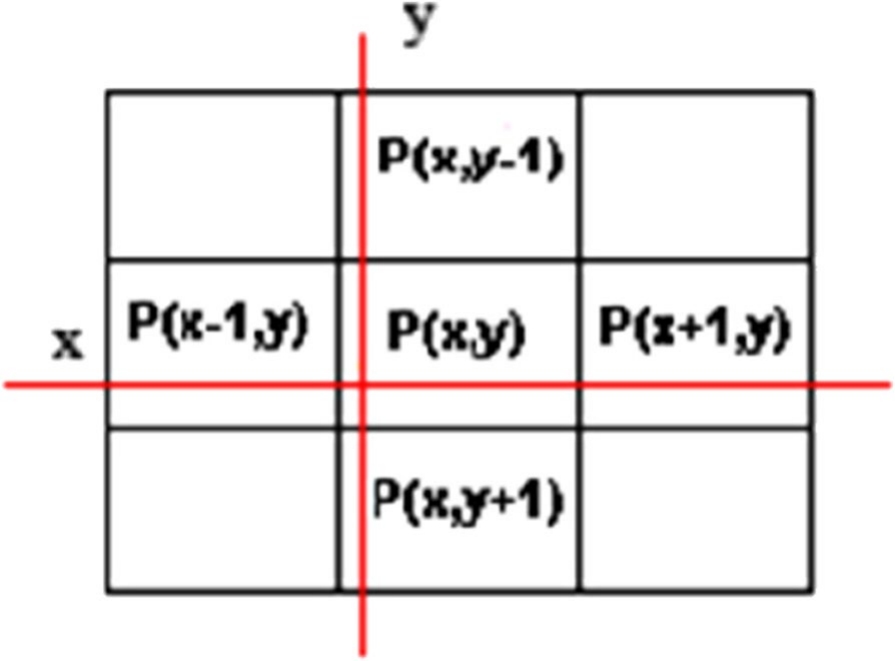
Braiding 8 adjacent points around the start pixel in a 2D image

5) The *points* command stores the first pixel coordinate in a matrix.

6) Based on Fig. 2, the color intensity of 8 adjacent pixels near the start pixel is controlled to be at the range of first pixel color intensity with suitable accuracy via *threshval* command, and is then supplemented by the following criteria for the *points* matrix:

2 $$\left|pointval\right|\le threshval+seedval$$

This method is called braiding.

**Fig. 3:**
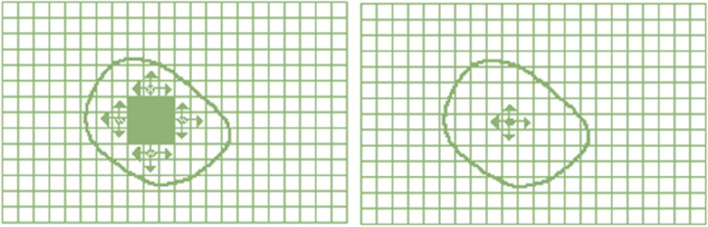
Applying primary area growth algorithm for 2D images

Another time, this criterion is controlled for adjacent pixles which are in a braid in the prior stage. Figure 3 shows this consecutive prodecure till the pixels towards the end of the braid are no longer qualified. At this momment, all selected pixels in the points matrix outline a surface as tumor scheme. The farthest pixels are selected as the tumor border, which is essentially curved.

**Fig. 4:**
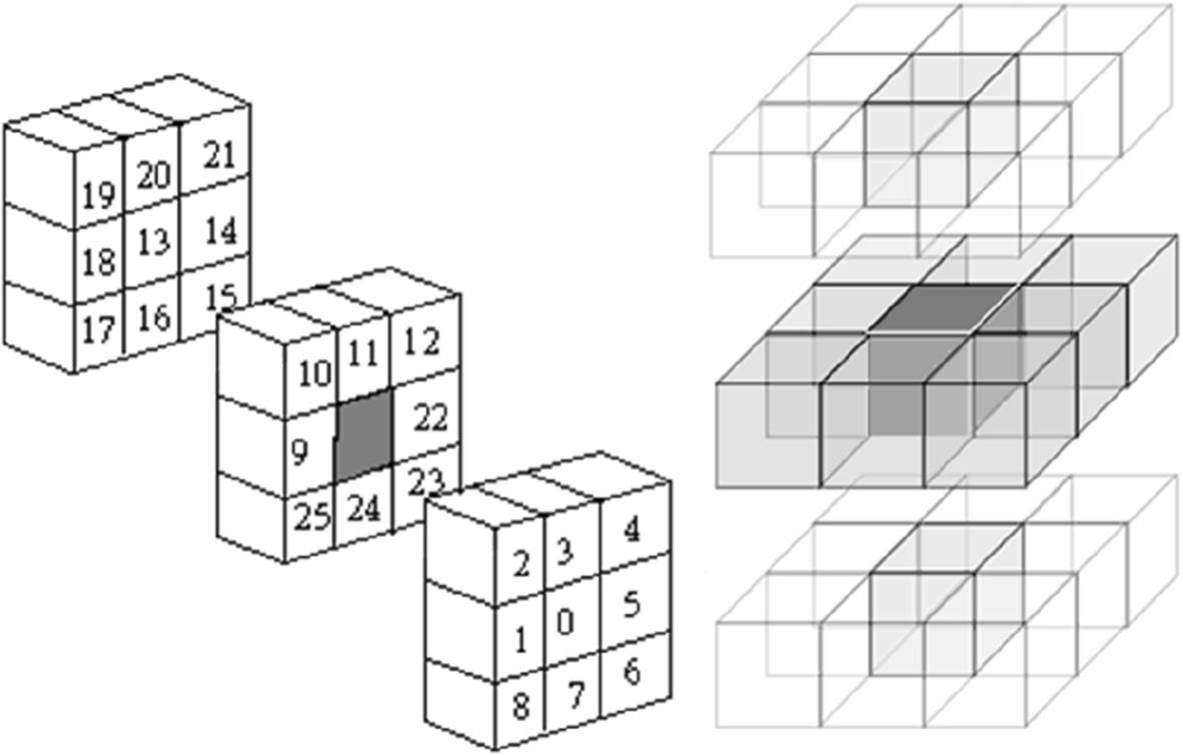
8 neighbor voxels (left), and region growing process (right) for 3D images

The primary algorithm for area growth of 3D images is started by selecting the coordinate point (voxel) by the user. All of the above steps are exactly the same for the voxels. Finally, according to Fig. 4, all the voxels in the *points matrix* that actually form a volume are considered to be tumor tissue. Also, the furthest voxels found are referred to as the tumor border, which actually form a shell.

Advantages of the primary area growth algorithm are: a) this technique can relatively accurate identify the tumor area; b) the speed of this method is much higher than other methods; c) little basic information is needed to start the algorithm; d) it is relatively simple to implement. Of course, this method also has disadvantages, which include: a) if the tumor is fragmented, it will not be able to identify all the fragments starting from just one point, and the tumor continuity is a prerequisite for this algorithm; b) in cases where the tumor is attached to the lung wall, the growth algorithm may result in an error and may cover some of the wall or even the outermost part; c) if the color difference between the tumor and the primary tissue is too small, the accuracy of the algorithm will be reduced.

### Enhanced Area Growth (EAG) algorithm

Here, a novel algorithm is presented to upgrade the area growth problems and control the regional protocol. The EAG method is divided into two parts: pre-procedure and main procedure for both 2D and 3D images.

#### Pre-procedure phase

##### Contrast augmentation

**Fig. 5:**
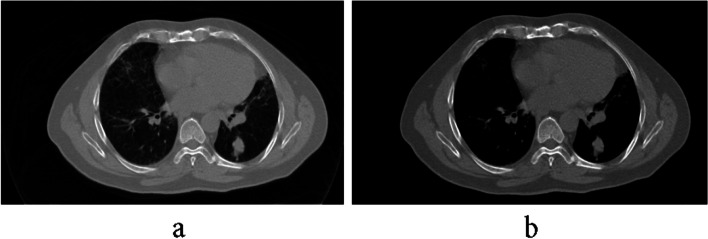
With (**a**) and without (**b**) contrast augmentation for better illustration

Provisionally, the increase in image contrast influences the detected tumor edge that decreases the accuracy of the provided segments. In this presented work, the primary tumor area is determined by primary algorithm, however, the contrast augmentation decreases the running time in the early stage. This augmentation brings about the veins to be white dots on the image and misguidedly diagnosed as a tumor, and then affects the growth of the area. Figure 5 shows the result of contrast augmentation in removing white dots for better illustration.

##### Appointing lung area

**Fig. 6:**
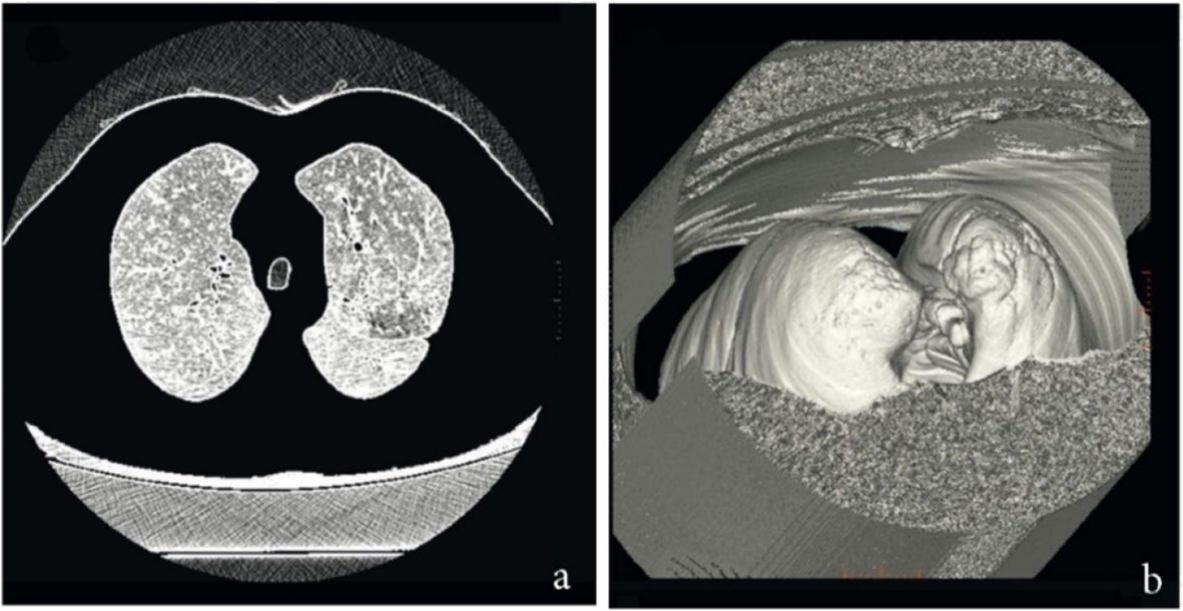
Appointing lung limit for (**a**) 2D and (**b**) 3D images

To distinguish between lung and tumor regions, the lung limit must first be determined by primary algorithm. This step prevents area growth outside the lung limit in cases where the tumor has become attached to the wall of the lung. Computed tomography images are illustrated in white and black colors, so that the color intensity is so large for neighbor segments. Therefore, applying primary algorithm besides contrast augmentation designates the lung limit precisely. On the other hand, morphological algorithms along with separate anatomy operations may also appoint the lung limit for the tumor designation. After the lung limit was appointed by *lungarea* command in MATLAB, a restriction is added to the primary algorithm in order to select the qualified points for tumor identification inside the defined lung limit. Figure 6 illustrates a case when appointing lung limit for 2D and 3D images.

#### Main procedure phase

##### Area growth constraints

**Fig. 7:**
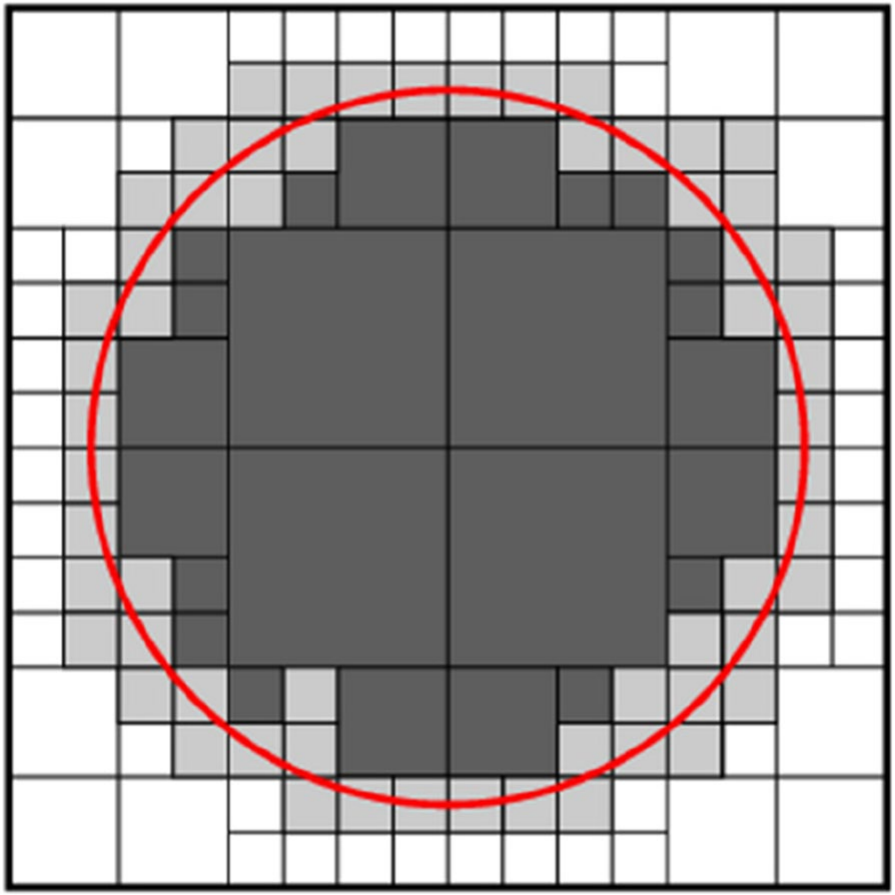
Area growth constraint established upon primary algorithm

Since maximum tumor volume is apparent during treatment, the algorithm may be constrained by considering the growth tumor area rather than the entire image. This organization reduces the running time of the algorithm. To set this constraint on the primary algorithm, the user selects a maximum diameter for the tumor so that the qualified points are at least one diameter far from the start point in growing. Indeed, the algorithm is confined to a circle in 2D input image and to a sphere in 3D image. Figure 7 shows this area constraint of interest in 2D mode.

##### Thresholding automatically

**Fig. 8:**
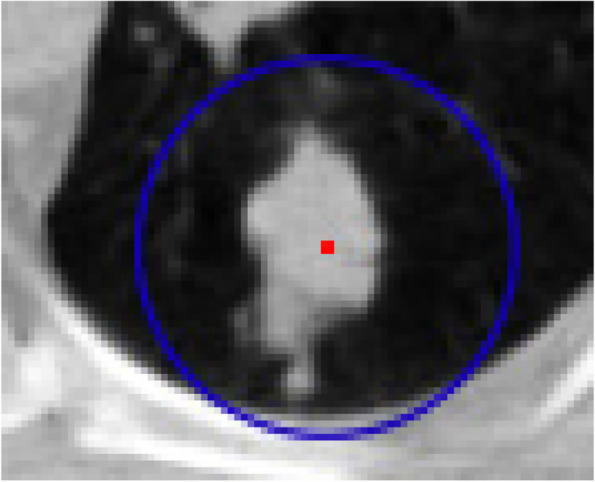
Automatically finding a local threshold

Automatically, the threshold was determined in order to check the neighboring points in 20% default of grey level threshold value of the input entire image as a minimum value by the primary algorithm. However the tumor borders are not fully realized, here, a 20% local threshold is defined as a new technique to boost the border identification. By proposed technique, the grey level thresholding is defined in the constraint area in where the tumor is placed. The color intensity of tumor center is determined so that this point is the same as the mass center in physics, except that the color intensity of each pixel or voxel plays a role in its mass. Therefore, the center of the tumor color intensity can be calculated from the following equations:

3 $$\begin{array}{l}{X}_{ic}=\frac{1}{{I}_{T}}\sum\limits_{k=1}^{n}I\left({p}_{i}\right)x({p}_{i})\\ {Y}_{ic}=\frac{1}{{I}_{T}}\sum\nolimits_{k=1}^{n}I\left({p}_{i}\right)y({p}_{i})\\ {Z}_{ic}=\frac{1}{{I}_{T}}\sum\limits_{k=1}^{n}I\left({p}_{i}\right)z({p}_{i})\end{array}$$

where n is pixel or voxel number of identified primary tumor, I_T_ is the sum of the color intensities of all the points mentioned, and I(p_i_) the color intensity of the point i. After finding the center of color intensity, its distance from the farthest border point of the tumor was calculated and then five units added to it. Afterward, in 2D mode by a circle and in 3D mode by a sphere with a radius of calculated value and center of color intensity is considered and subsequently this area used to determine the new threshold. That is, the new threshold limit is defined as 20% of the grey threshold of the tumor area and the primary algorithm is applied starting from the previous start point with this new threshold. Figure 8 shows an example of how the threshold is determined automatically.

##### Definition of comparison quantity

At all stages of the primary algorithm, the color intensity of the local points is examined with the start point value. If we move away from the start point, there is a possibility of an error. In order to increase the accuracy and decrease the error, it is suggested that the area growth of *the comparison quantity* be modified at each level of the algorithm to the mean of the points recognized as tumors up to this level. Up until now, if the number of points recognized is n points, the *comparison quantity* is modified as follows:

4 $${seedval}_{modified}=\frac{1}{n}\sum\nolimits_{i=1}^{n}I({p}_{i})$$

and I(p_i_) is the color intensity for point i.

##### Area growth from several start points

**Fig. 9:**
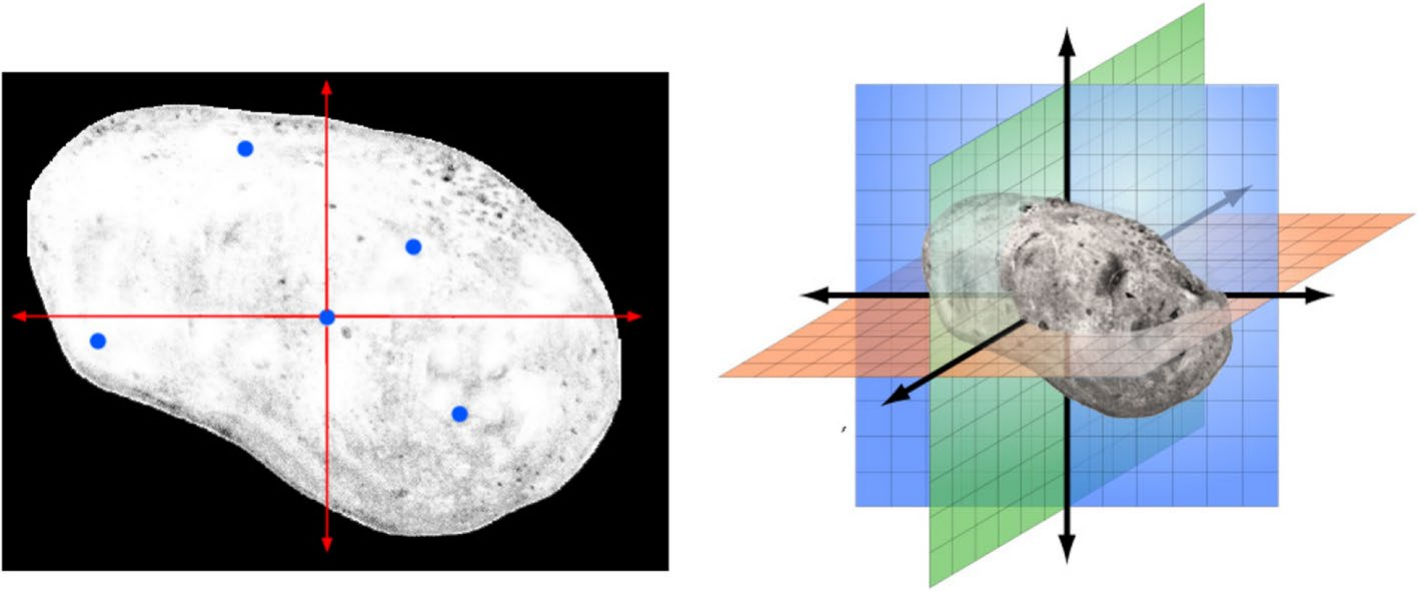
Designating different start points in 2D and 3D modes towards enhanced area growth

Given that the start point of the algorithm is determined by the user, it is obvious that every time we run the primary algorithm and start from diverse points, different results are achieved. In order to improve the accuracy of the final result, it is suggested to run the growth algorithm starting from several dissimilar points. Nonetheless, the user still determines simply a start point, and the other points are rationally chosen from the total tumor volume or surface in the following distinct rout. In the meantime, the independence of the final result from the start point is largely ensured. This route is:

1. By running the primary algorithm on the start point determined by user, the geometric center of the tumor is settled (Obviously in 2D mode there is no need to calculate z.),
  5 $$\begin{array}{c}{X}_{center}=\frac{1}{number\;of\;voxels}{\sum }_{(x,y,z)}x\\ {Y}_{center}=\frac{1}{number\;of\;voxels}{\sum }_{\left(x,y,z\right)}y\\ {Z}_{center}=\frac{1}{number\;of\;voxels}{\sum }_{(x,y,z)}z\end{array}$$
2. By this new coordinate, the tumor is divided into 4 and 8 sectors for 2D and 3D modes, respectively. Accidentally, a point is selected in each sector.
3. The primary algorithm starts with 5 points in 2D mode and 9 points in 3D mode, where one of them is the center of the tumor.
4. The specified new-fangled tumor comes from the interpolation between the tumors established in the previous stage. Figure 9 shows the designating different start points for enhanced area growth algorithm in 2- and 3-dimensional growth.

##### Improvement of tumor edges

**Fig. 10:**
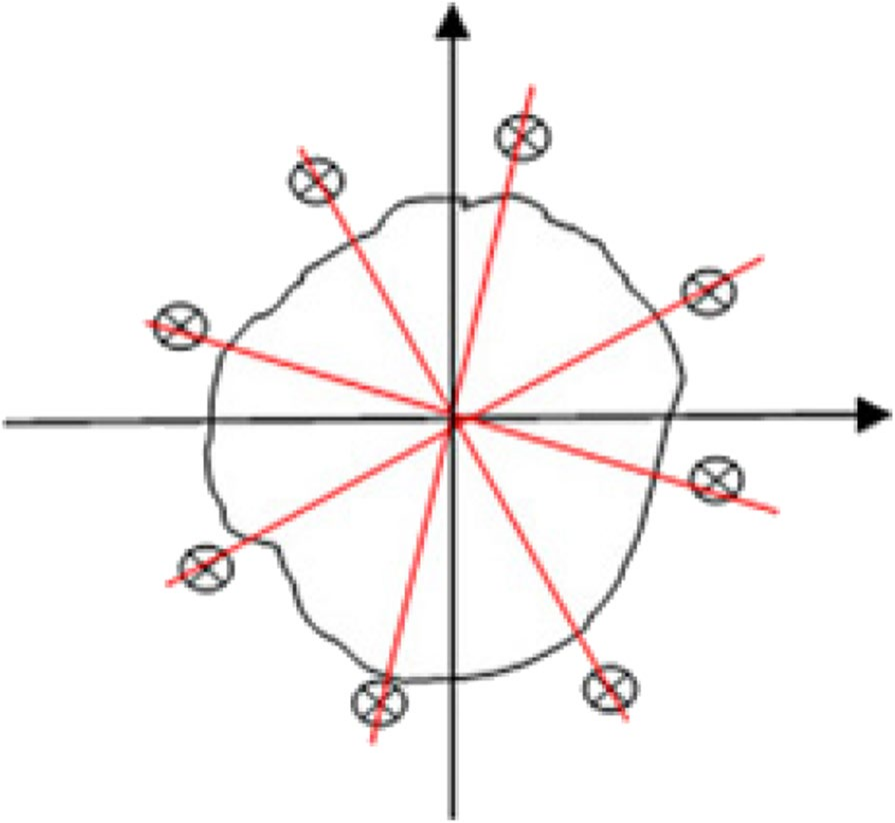
Edge improvement process in 2D mode by introducing new points around the primary tumor segment

In some cases, after finding the border of tumor, the relevant edges are not completely distinguished. So, the edges are improved in the last step by the proposed algorithm. This edge improvement procedure partitions the entire angle of view to eight subsections for 2D mode and the entire solid angle to 16 subsections for 3D mode. In that case, one point is selected from each subsection by chance. Afterwards, three units go off in different directions from the tumor center toward start points on the edges derived from primary algorithm. Here, thresholding is utilized by 10% in place of 20%. Then, implementation of EAG algorithm on these new points generates a new subsection which can be appointed as follows: the new subsection is thrown out if: **i)** its median is less than the primary tumor median minus three times the standard deviation, ii) its surface ratio in 2D or volume ratio in 3D to the primary tumor section is greater than 0.2; or else the new subsection is joined to the primary tumor section. Figure 10 shows a schematic of the algorithm in 2D mode to come up an enhanced algorithm.

#### Enhanced area growth algorithm

The stages of the enhanced algorithm for 2D mode are optimized as follows:

i) After calling lung 2D image, the contrast is augmented.
ii) By MATLAB, the *lungarea* command is appointed on the anatomy and the pixel of start point designated by the user. Then, *seedval* command saves the primary value based on its color intensity.
iii) *radiusval* command is utilized to define the maximum radius of the specified tumor.
iv) *threshval* command is used for the entire input image by 20% grey threshold.
v) The first pixel coordinate is saved in *matrix points*. Next, eight surrounding pixels are inspected to see their color intensities are within the range of the first start point inside the determined lung area of stage ii. Besides, their distances are no more than twice as large as the maximum radius anticipated in stage iii. All these points are inserted into the matrix if the *point value* is between the *primary (start) value* ± *the threshold value*.
vi) The primary or start value is modified to the points’ median based on the prior stage in the braid.
  6 $${seedval}_{modified}=median\;({I}_{points})$$
vii) These modified surrounding pixels are re-inspected under stage v circumstances to generate new points values. This route is valid till the pixels are no longer qualified and the braiding is finished. Therefore, all points in the matrix shape a shell as tumor border.
viii) The maximum distance between primary (color intensity center) and new tumor borders is estimated. By defining a circle with the primary tumor border and adding 5 units to the estimated maximum distance, another new tumor area is created. Again for this area, new threshold is 20% of the grey threshold value.
ix) The stages v to vii are re-performed by selecting the start point, and then the new threshold is dedicated in the stage viii to estimate the new tumor border.
x) The tumor is partitioned to four subsections from the center point and a point is accidentally selected within each subsection. Then, stages v to ix are re-performed for these five points in order to specify the new tumor border via interpolation between these five points. Subsequently, the eight lines at different angles (0, 45, 90, 135, 180, 225, 270 and 315 degree) from the center point are drawn to delimit the points of intersection with the tumor borders. Afterwards, it’s three units away from the boundary, with eight points for edge improvement.
xi) Once more, stages v to vii are repeated by considering these new eight points with 10% threshold, and partitioned to eight growth areas. Each subsection is linked to the main area by satisfying two conditions: {median (new area) > median (primary tumor) – 3*standard deviation (primary tumor)}, and the ratio of new surface to primary tumor surface is less than 0.2. Eventually, the obtained surface is delineated as a segmented lung tumor.

**Fig. 11:**
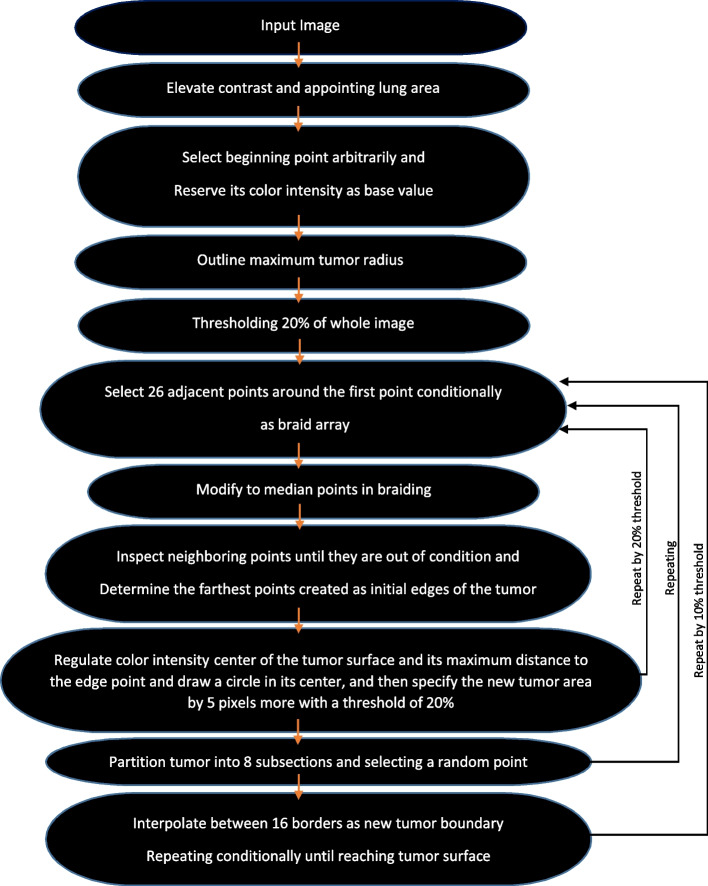
Flowchart of Enhanced Area Growth algorithm for 3D images

According to Fig. 11, most stages of the enhanced algorithm in area growing for 3D images are as 2D ones, in which a 3D image is first uploaded and the coordinate of the start point (voxel) is determined by the user. By stage v, the braiding is performed for 26 voxels considering corresponding conditions. Meanwhile by stage x, the tumor center is partitioned to 8 subsections and we have a total of 9 points via selecting one point in each subsection by chance. After finding 9 tumor boundaries and interpolation between them, the new tumor boundary is determined to achieve 16 points for edge correction via intersections with the tumor boundaries at angles of -180, -90, 0, 90, and -90, -45, 0, 45 degrees from the tumor center. At last, these obtained 16 new points with a threshold of 10% partitioned to 16 grown areas. Considering the conditions in stage xi, we will finally reach the segmented tumor in 3D mode.

### Lung CT images

Three official websites — LCA Laboratory (Lung Cancer Alliance) [22], DIR Laboratory (Deformable Image Registration) [23], NSCLC (non-small cell lung cancer) from the Cancer Imaging Archive (TCIA) Public Access [24], and LIDC (Lung Image Database Consortium) [25] — were utilized as reference ratings to evaluate the presented algorithm on lung tumor CT images. No permissions are required for use of the data and it is publicly available on these laboratory websites. Occasionally, image formats are different from the accessible information. In order to integrate the EAG algorithm besides the comparability of the outcomes, all formats and numbers were doubled and normalized to 1 and 0. Table 1 shows these images for both males (M) and females (F). All images have been taken with CT in DICOM format and also contain a tumor.

**Table 1:** The lung CT images utilized from DIR, LCA, NSCLC, LIDC labs [22–25]

Laboratory	Age and sex	Size of image	Number of image
DIR	Dissimilar ASL	Different dimensions	1–5
LCA	Dissimilar ASL	Different dimensions	6–10
NSCLC	Dissimilar ASL	Different dimensions	11–30
LIDC	Dissimilar ASL	Different dimensions	31–60

All MATLAB implementations were done with Intel Core 2 Duo T6670 / 2.2 GHz processor. It was executed for both 2D and 3D images, but the images shown are mostly 2D except for the final algorithm which also shows 3D images.

### Statistical analysis

In this survey, Dice coefficient was also utilized to assess the performance of the segmentation algorithms quantitatively. Given A as a segmented structure, B as a ground truth structure, and |*| which signifies the size of a binary set, hence the Dice coefficient [26, 27] C_dic_ is presented as:

7 $${C}_{dic}=\frac{2\left|A\cap B\right|}{\left|A\right|+\left|B\right|}$$

This coefficient denotes the ratio of the overlapped area between the segmented area and the truth area (0 ≤ C_dic_ ≤ 1). Its maximum amount is 1 when the segmented area is identical to the truth area, and its minimum amount is 0 when the segmented area completely misses the truth area.

To analyze the statistical behavior, a Student t-test was performed via assuming a normal distribution for two independent samples of the metric amounts. The p-value was calculated from 60 images using the acquired metric amounts. The data point numbers were enough large to undertake a normal distribution for the recorded mean metric for both algorithms supporting the t-test. Also, a significance level of 5% was assumed to indicate a major difference between the algorithms performances for a specified metric.

## Results

### Contrast augmentation

**Fig. 12:**
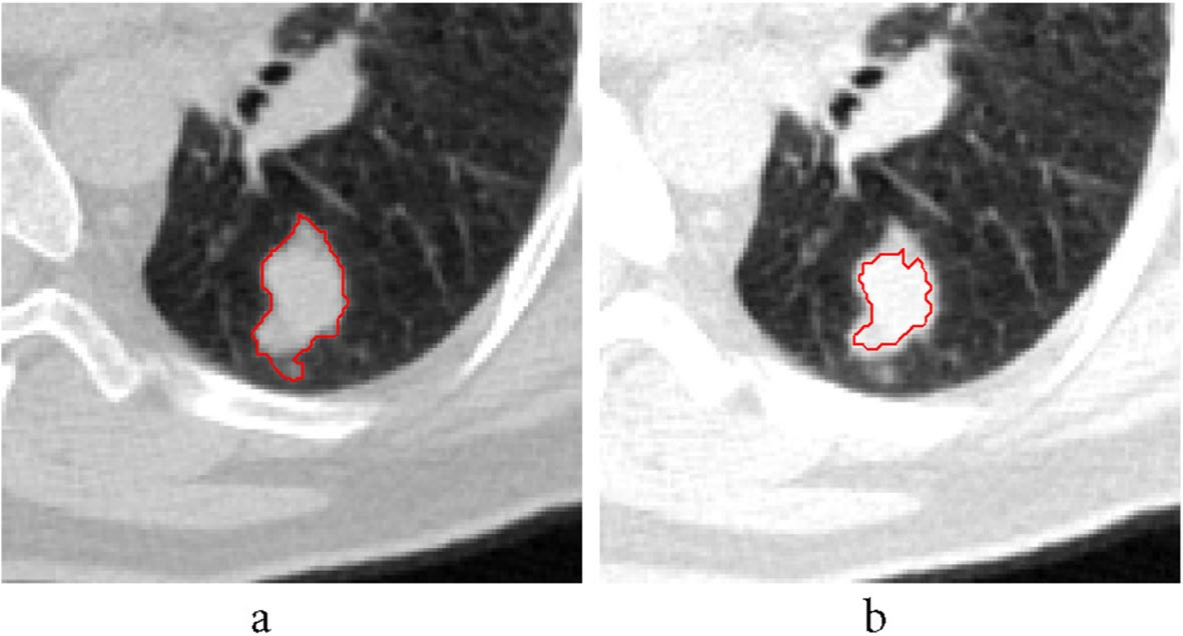
Implementing a temporary increase in contrast: **a**) without **b**) with increasing contrast

First, a contrast improvement was made for each image in implementation. *Imadjust* command was utilized to boost the contrast in 2D mode. Since there is no ready command in MATLAB for 3D mode, it was defined a function as *imadjust3d* to increase the contrast. Figure 12 shows an example of contrast augmentation and its effect on the result of the primary algorithm. As the contrast increases at first, a clear image of the designated tumor is obtained. In Table 2, the differences in obtained results have been given. By increasing the contrast, the number of pixels decreases and processing time also reduces.

**Table 2:** The average data applied for DIR, LCA, NSCLC, and LIDC labs

Status	The number of pixels specified	Elapsed time (s)
without increasing contrast	994	0.35
with increasing contrast	566	0.28
Time saving	–	20%

### Appointing lung area

**Fig. 13:**
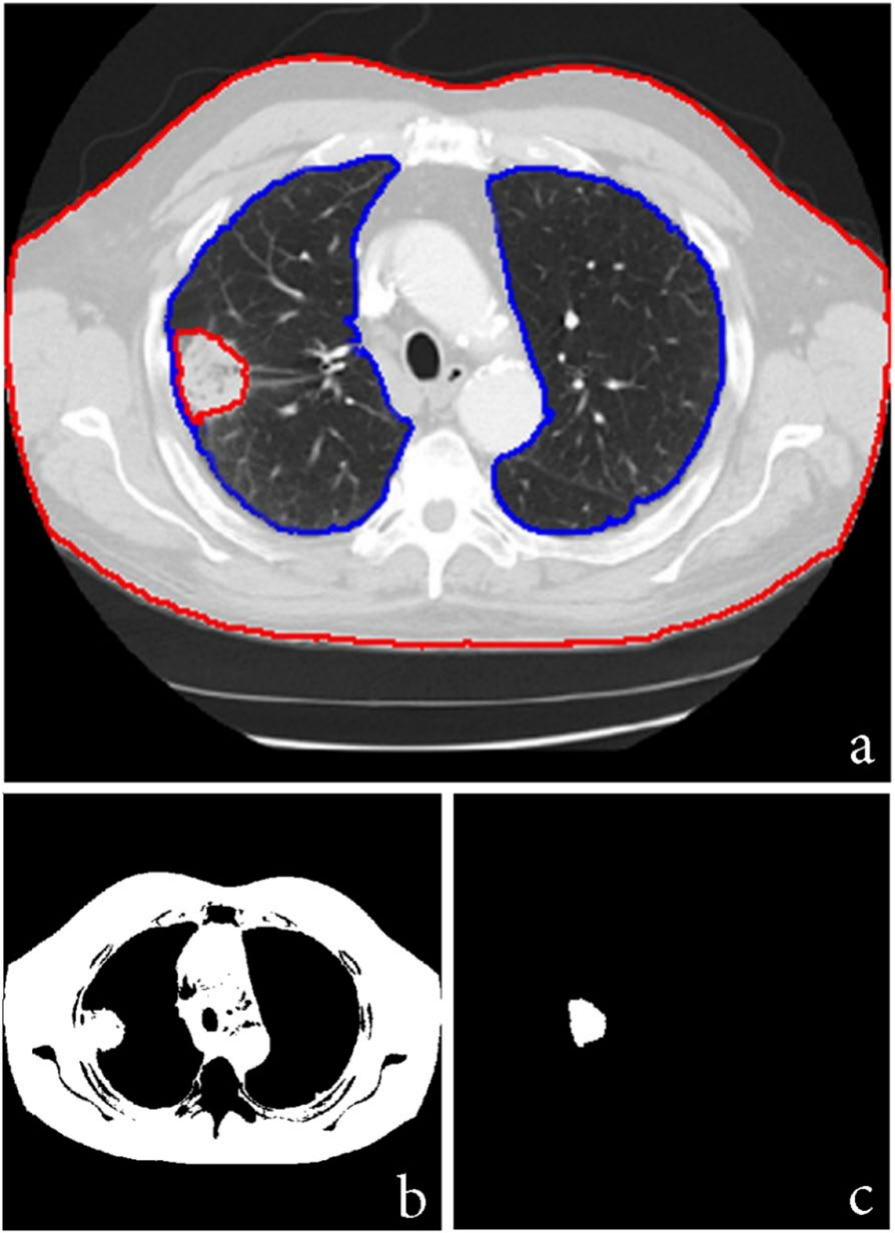
Appointing lung area before applying the algorithm: **a**) with **b**) without segmentation. **c** Result of finding lung tumor area with segmentation at the beginning of the algorithm

In many cases it has been observed that the tumor attaches to the lung wall, making it difficult to detect the tumor accurately. For this reason, the lung area is first determined. Figure 13 shows the result of specifying the lung area before applying the area growth algorithm. As can be seen in Fig. 13b, failure to specify the area has led to a major error in process of the growth algorithm and its interference in the lung wall. But in Fig. 13c, the algorithm is successfully implemented by specifying the lung area at the beginning of the process.

**Fig. 14:**
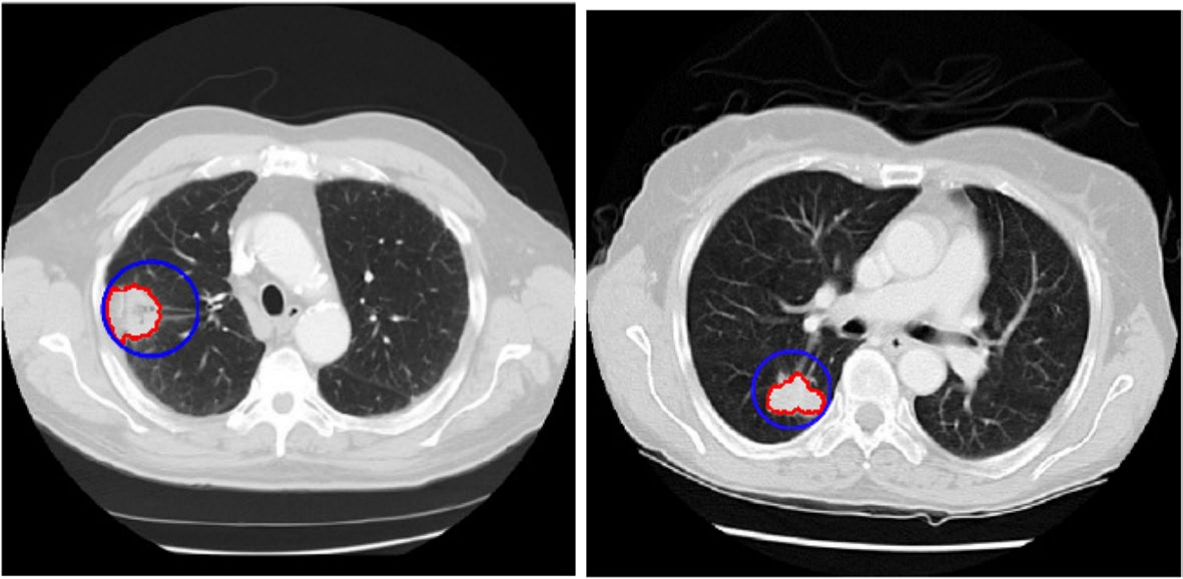
The result of the constraint on growth area by radius of 70 (Left) and 50 (Right) units

On the other hand, constraint in growing area can be helpful in better diagnosis. Apparently by Fig. 14, this constraint has hindered the area from misguidedly joining the lung wall. The maximum expected radius for the user-defined tumor is 70 and 50 units for patients 1 and 2 (Table 1), respectively.

### Discerning threshold automatically

**Fig. 15:**
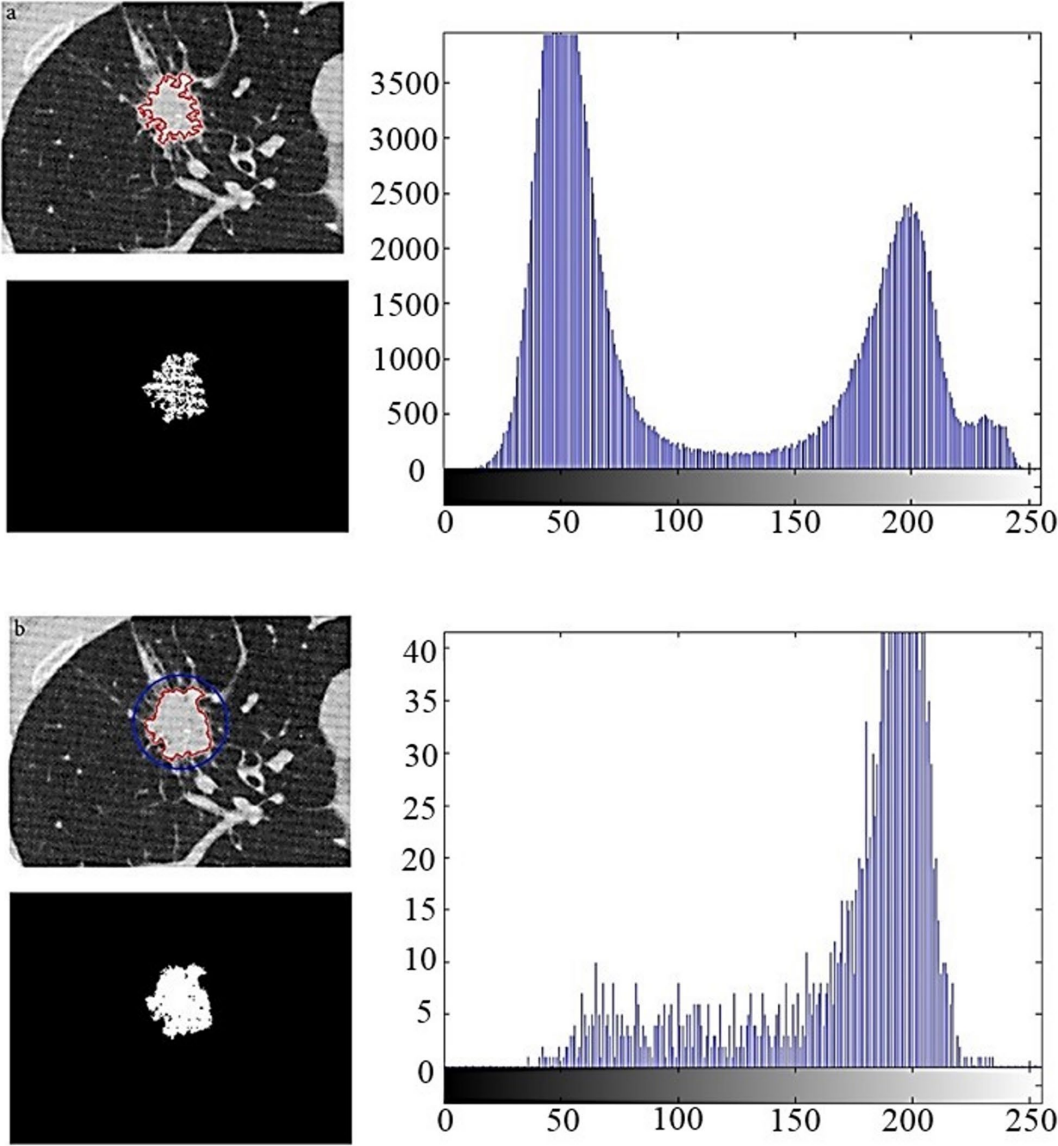
Discerning threshold values for: **a**) preliminary and **b**) automatic styles

Figure 15 shows an example of execution of the automatic threshold recognition technique. Figure 15a refers to a circumstance where the preliminary threshold value was totally considered 20% of the grey threshold. Here, the grey threshold is 0.48 and threshold amount is 0.096. Following discerning initial tumor, the coordinate of the color intensity of the tumor was appointed and 34 units were detected for the maximum distance from this tumor center. Then, a circle with 5 radius units was depicted surrounding the tumor as shown in Fig. 15b. In this situation, the 20% threshold is 0.128 and the grey threshold is 0.64. Moreover, the histogram of the whole image in Fig. 15a and the *targetarea* histogram in Fig. 15b show the shift of the peak to 1, namely the bright colors, which also show the grey threshold shift.

**Fig. 16:**
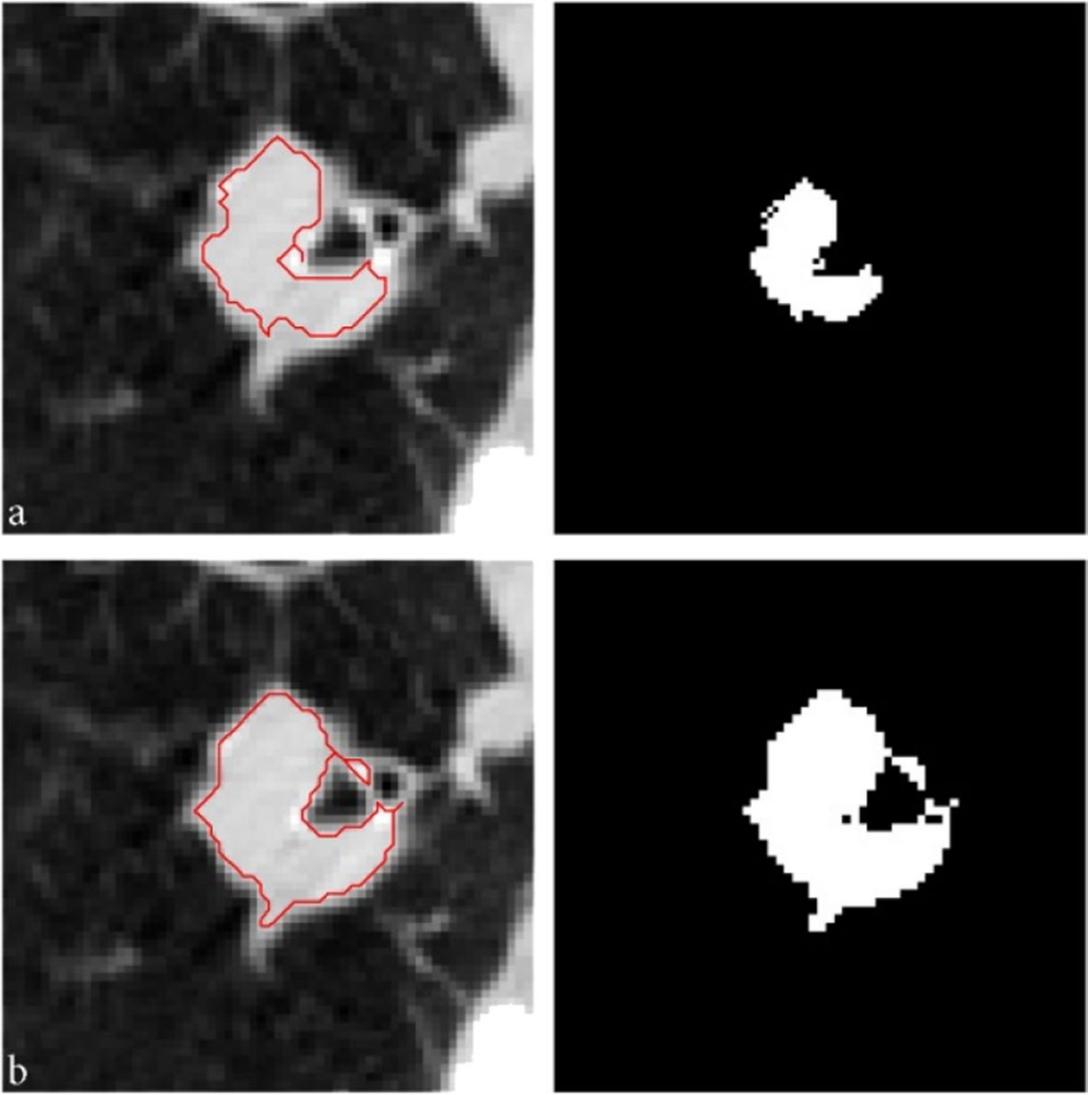
Obtained images from *comparison quantity* of color intensity for **a**) preliminary and **b**) modified implementation

**Fig. 17:**
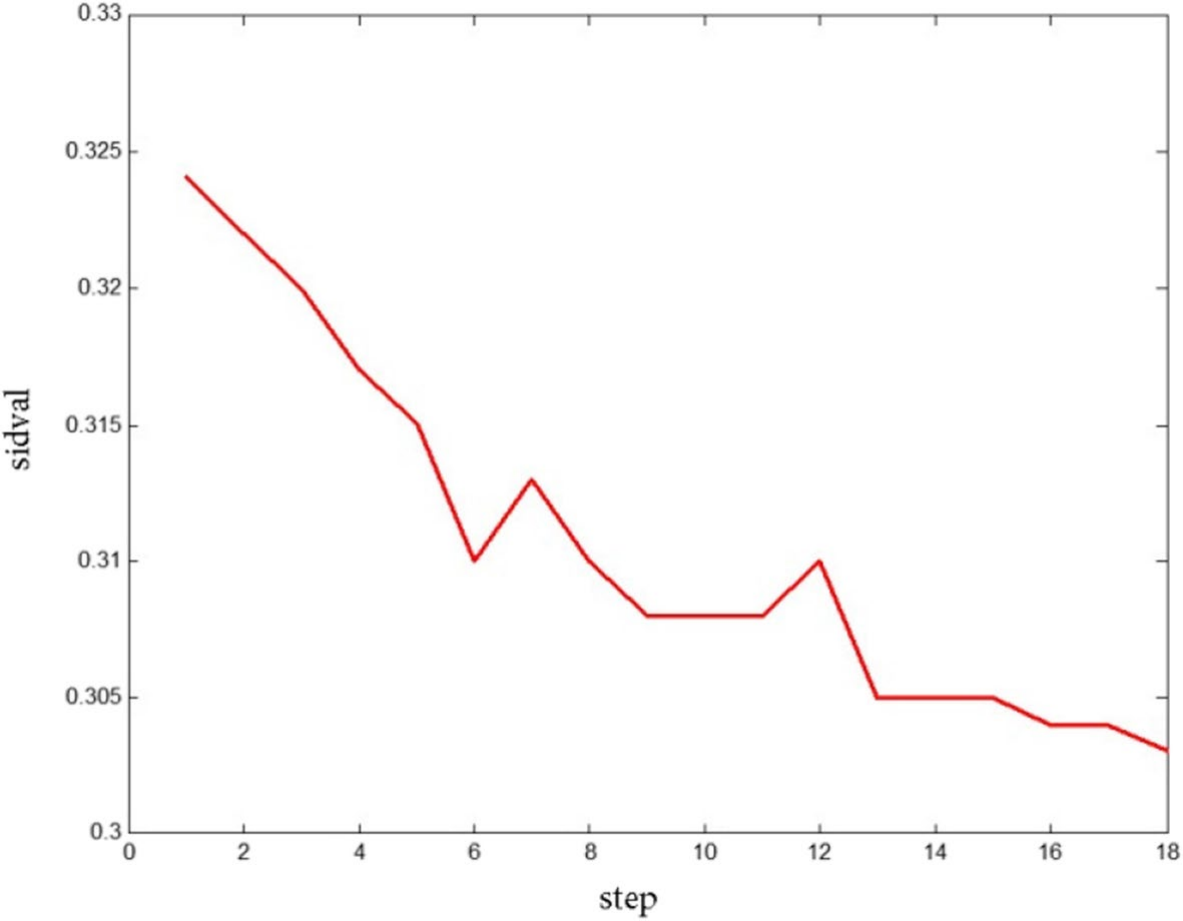
Color intensity variations at different steps of the algorithm in automatic thresholding

Furthermore, in this research, modifying the *comparison quantity* of color intensity (Eq. 4) has been applied at each step of the algorithm. According to Fig. 16 by this modifying, the image was improved and the tumor edges were also better covered. Also, Fig. 17 shows the *comparison quantity* alterations at different steps that decrease the color intensity as the steps progress.

### The results of growth from various points

**Fig. 18:**
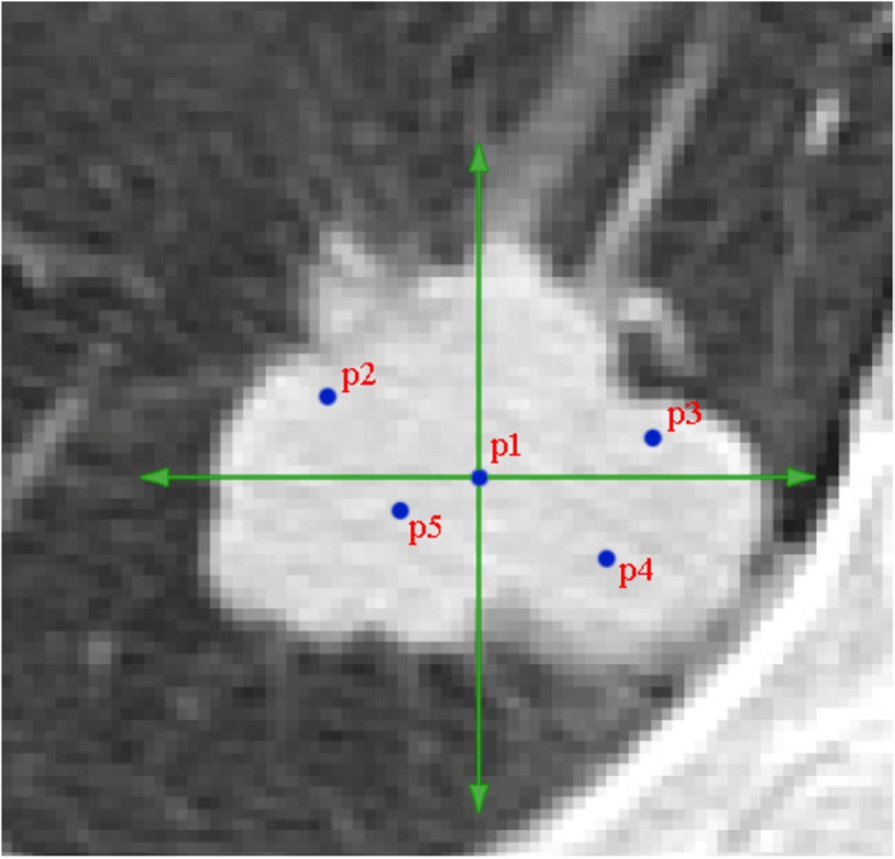
Selected points in initial tumor to begin growth from several points by p1 center position of (180,334). The other (x,y) locations are: p2 (165,328); p3 (196,329); p4 (192,342); p5 (173,344)

Figure 18 shows the performing of the growth beginning from various points. After the primary tumor was appointed, the geometric center of the tumor is resolved by the point p1. Then, the tumor is partitioned to four subsections by the p1 center to determine p2 to p5 points, the locations of which are shown in parenthesis.

**Fig. 19:**
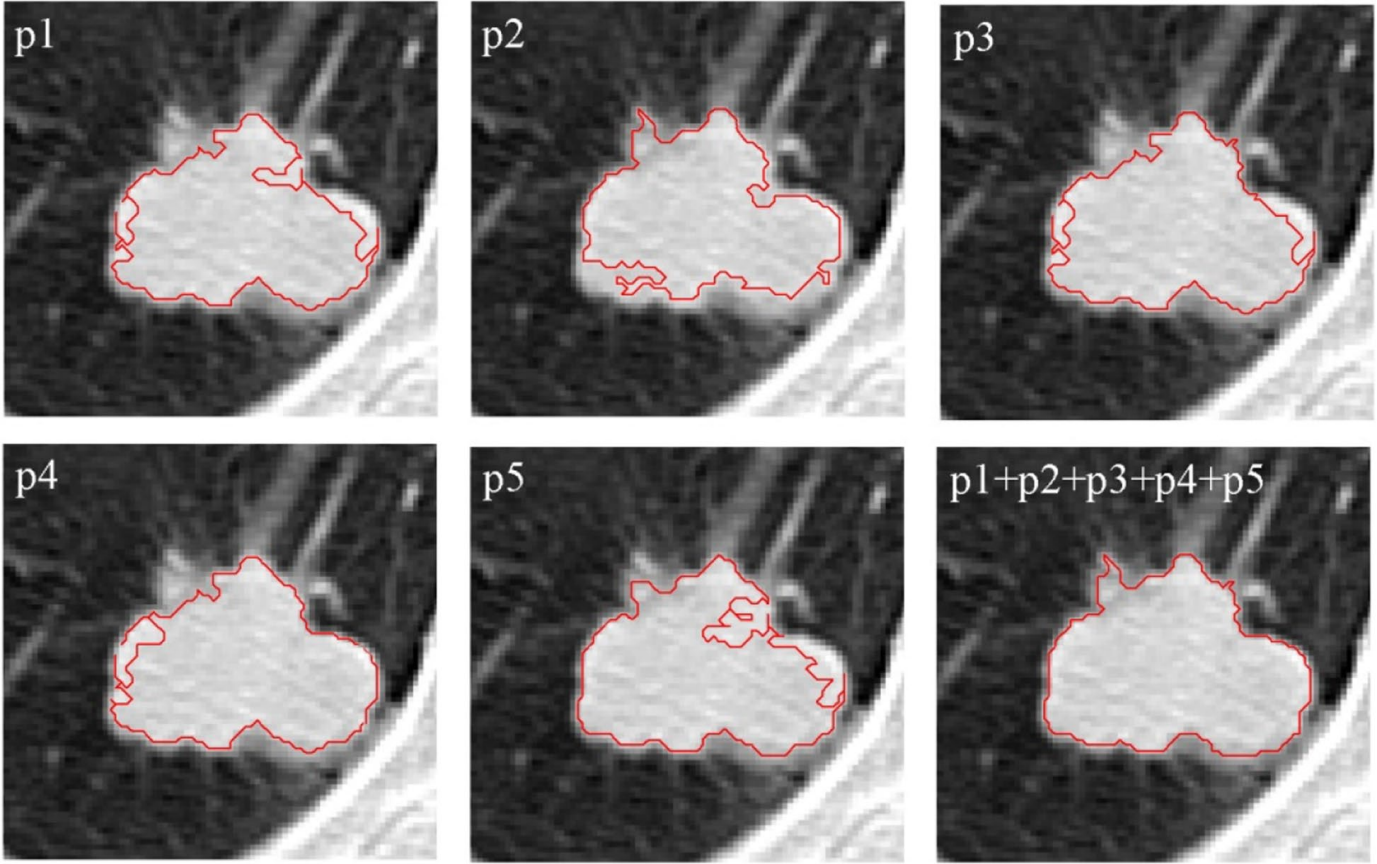
Growth from various points of Fig. 18 to delineate the tumor area

This growing continues surrounding each of the five points and the outcomes have been displayed individually in Fig. 19. The final image merges the multiple growing to create a more accurate image of limiting and delineating the tumor area.

**Fig. 20:**
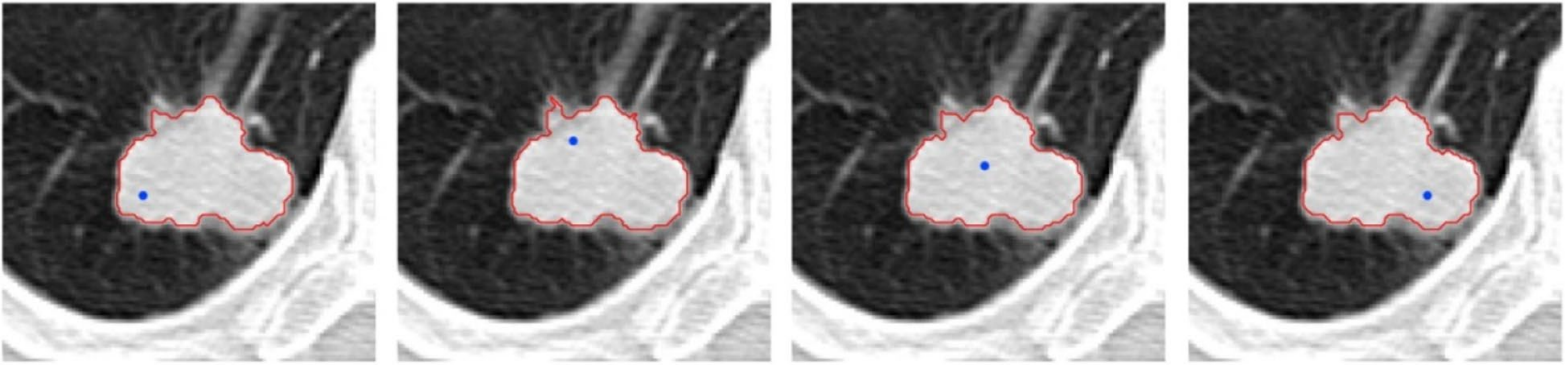
Independence of the growth algorithm result from the start point

The method presented, growth starting from several points, guarantees the independence of the final result from the start point. Despite the start from different points, Fig. 20 shows that the obtained image is independent of the start point. The start point appointed by the user has been shown in each image.

### Improvement of tumor edges and EAG Algorithm

**Fig. 21:**
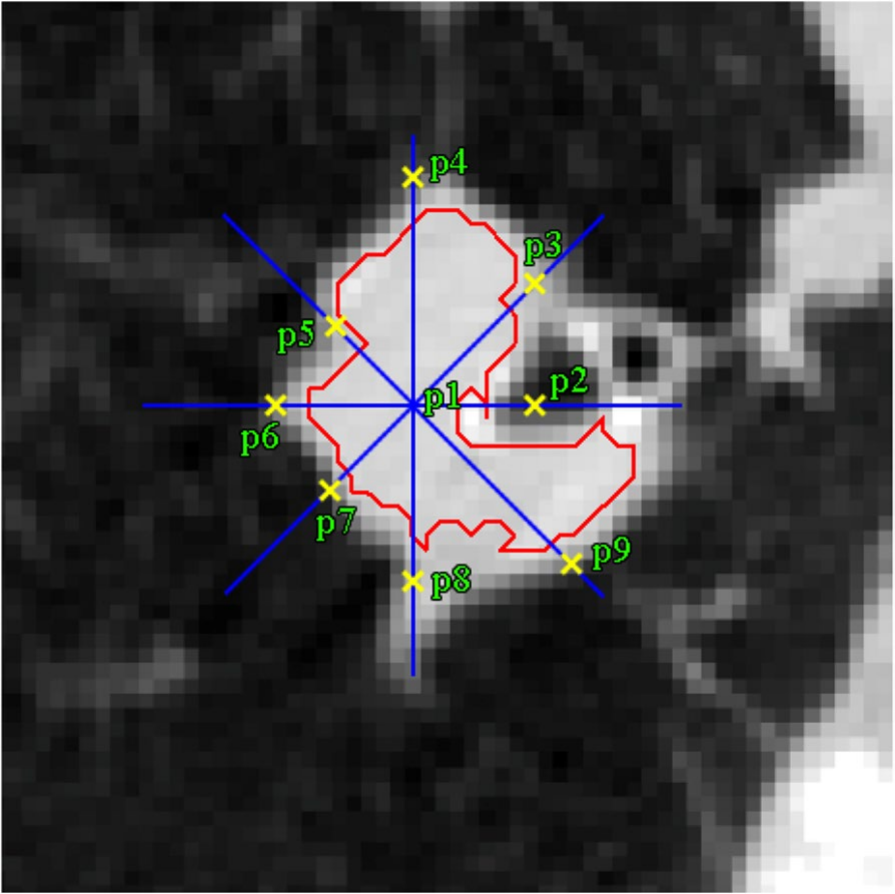
Nine detected points to improve the tumor edges

**Fig. 22:**
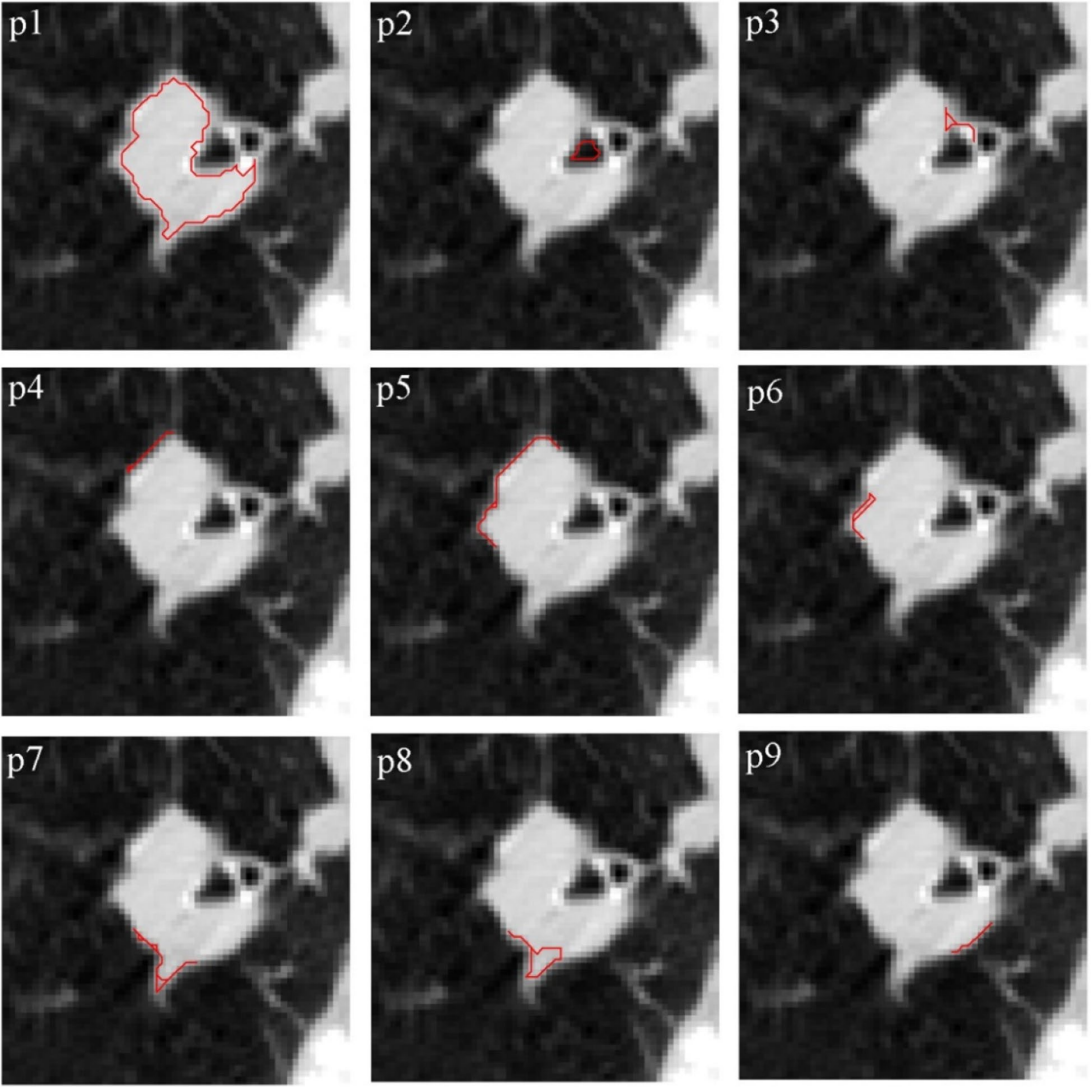
Region growth result at selected points on the edges

By determining the center point in 2D mode, eight points are chosen with the same angles and three pixels’ distance from the tumor edge. A total of nine points are candidates to improve the edges and grow the area by 5% threshold, as shown in Fig. 21. Each designated point identifies a section around the edge and is validated in accordance with the conditions set out in the Methods section. The growth results from each of these points have been shown in Fig. 22.

Also the coordinates of the chosen points on the edges and the accuracy of their result have been shown in Table 3.

**Table 3:** The coordinates of the selected points to improve the edges and their accuracy

Point	Coordinate (x,y)	Accuracy
P1	(219,250)	yes
P2	(281,250)	no
P3	(280,188)	yes
P4	(219,132)	yes
P5	(177,209)	yes
P6	(146,250)	yes
P7	(175,193)	yes
P8	(219,340)	yes
P9	(300,331)	yes

**Fig. 23:**
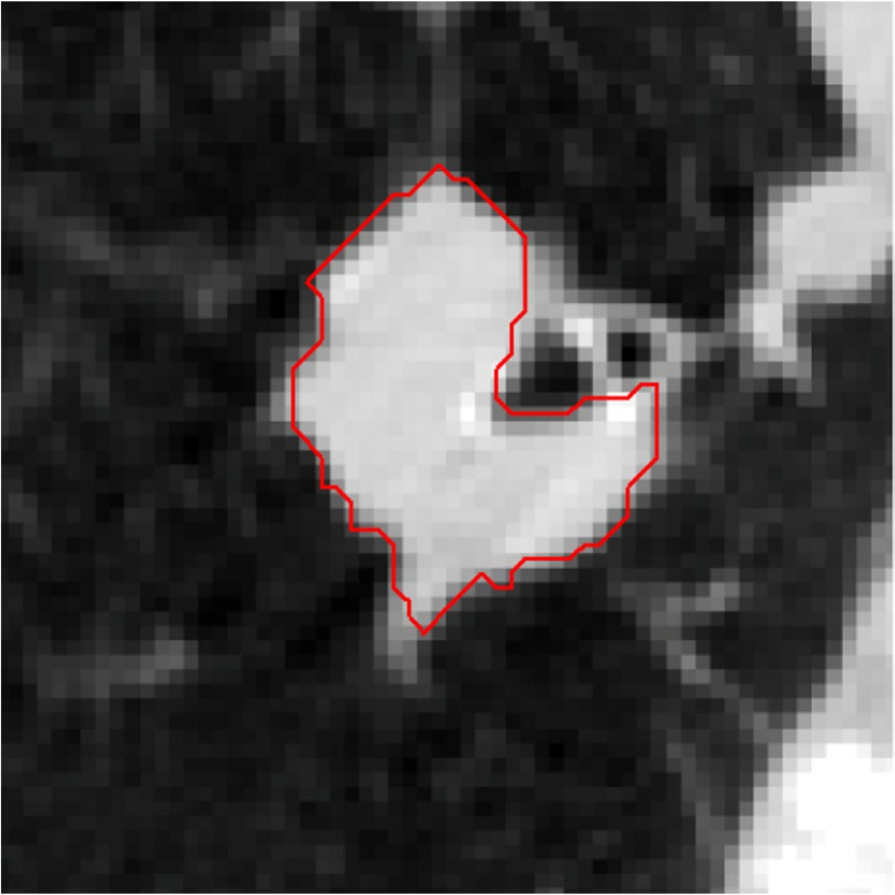
The final image from edges improvement by EAG algorithm

Only a few specified points on the edges are suitable for creating new subsections and can then be joined to the primary area, as shown in Fig. 23 by segment acquired.

**Fig. 24:**
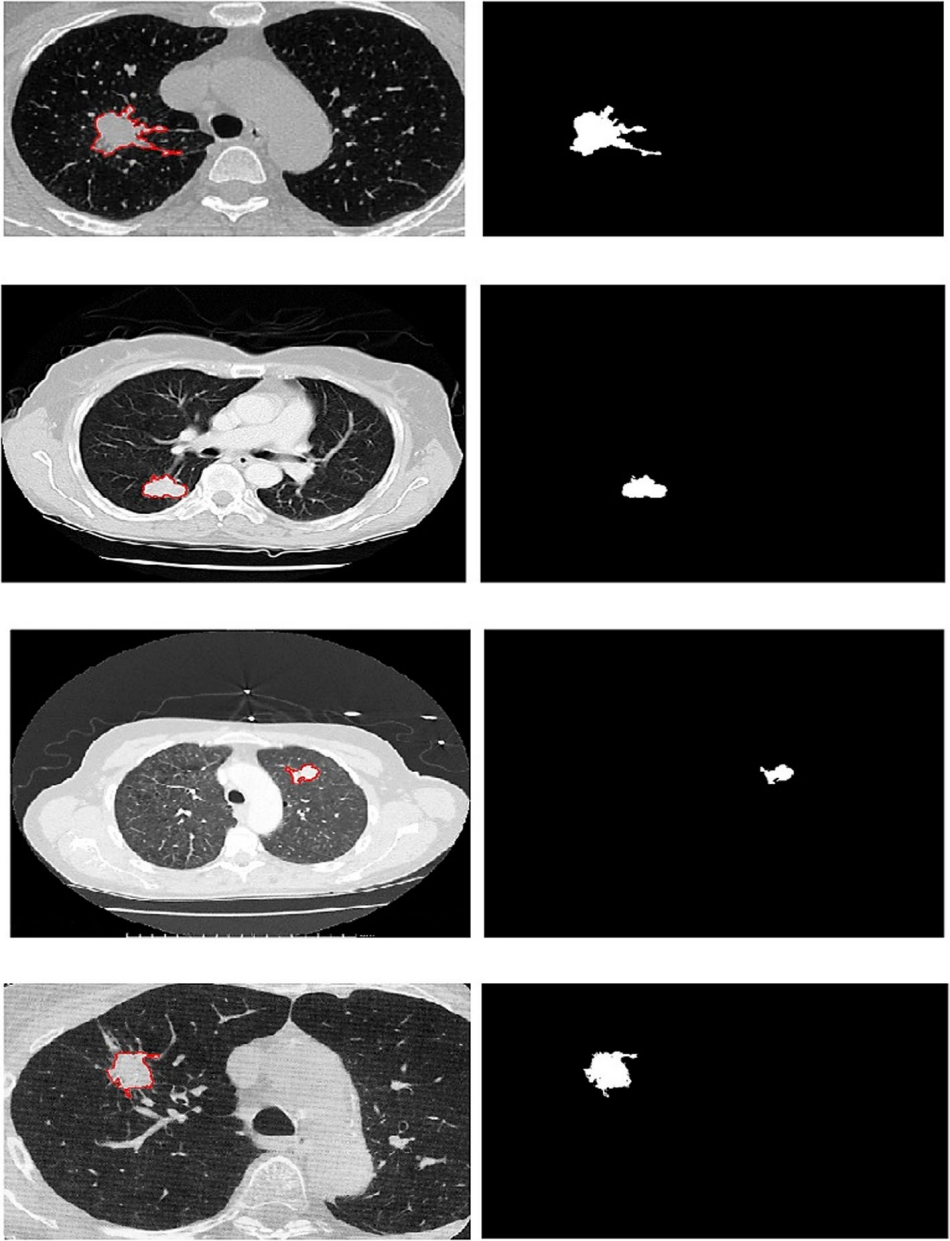
Implementation of EAG algorithm on 2D images

**Fig. 25:**
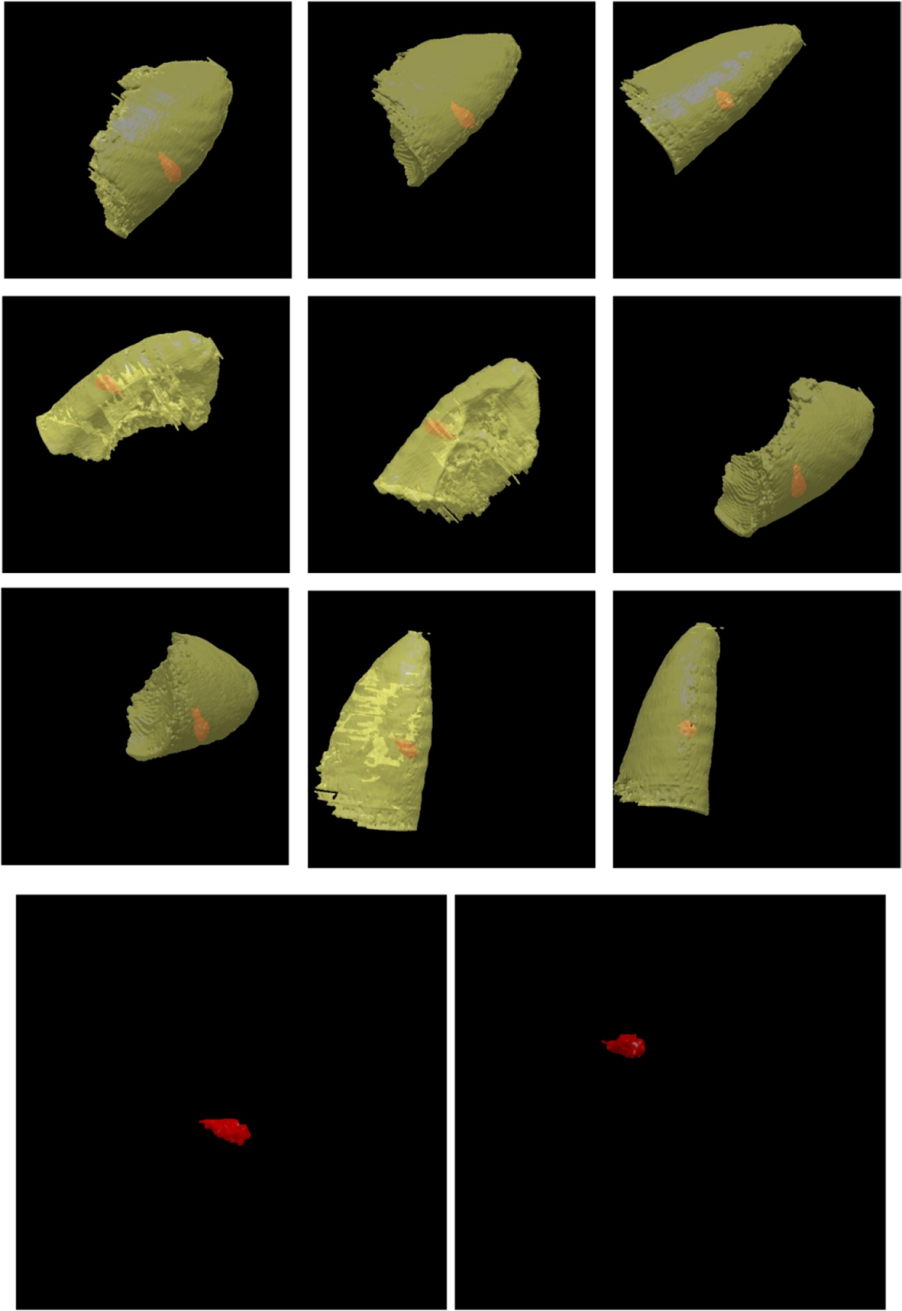
Implementation of EAG algorithm on 3D images. The lung and tumor regions are shown in yellow and red, correspondingly

For instance, the final results of the EAG algorithm when applied to 2D and 3D images have been illustrated in Figs. 24 and 25, respectively.

### Area growth process

**Fig. 26:**
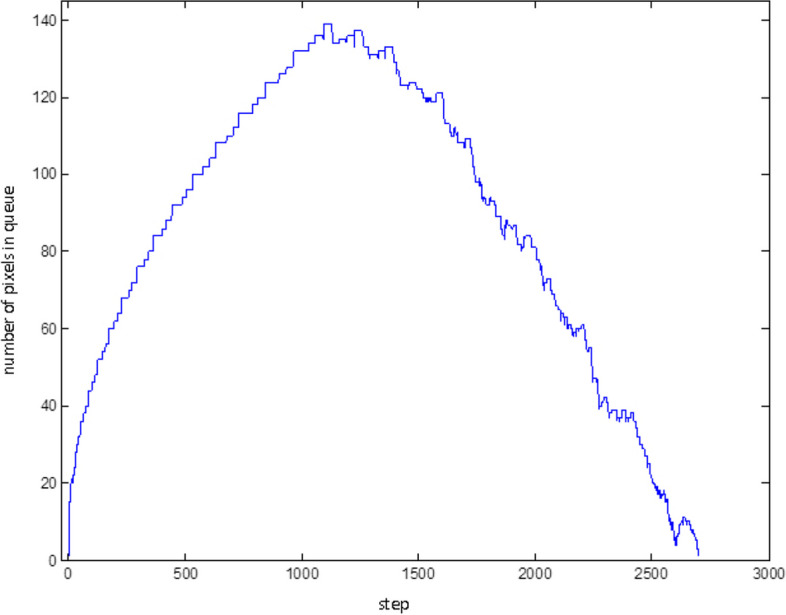
Number of pixels in the braid at consecutive steps of the algorithm to check for tumor discretion

Figure 26 shows the number of pixels in the braid examined and not yet qualified for the tumor points. At the beginning of the graph, the pixels in the braid are increasing until no more new pixels are braided and the chart starts to sink. As long as all the target pixels are examined, the chart reaches zero. The fluctuations in the descending part of the chart show the extent of roughness on the outer edge of the tumor.

**Fig. 27:**
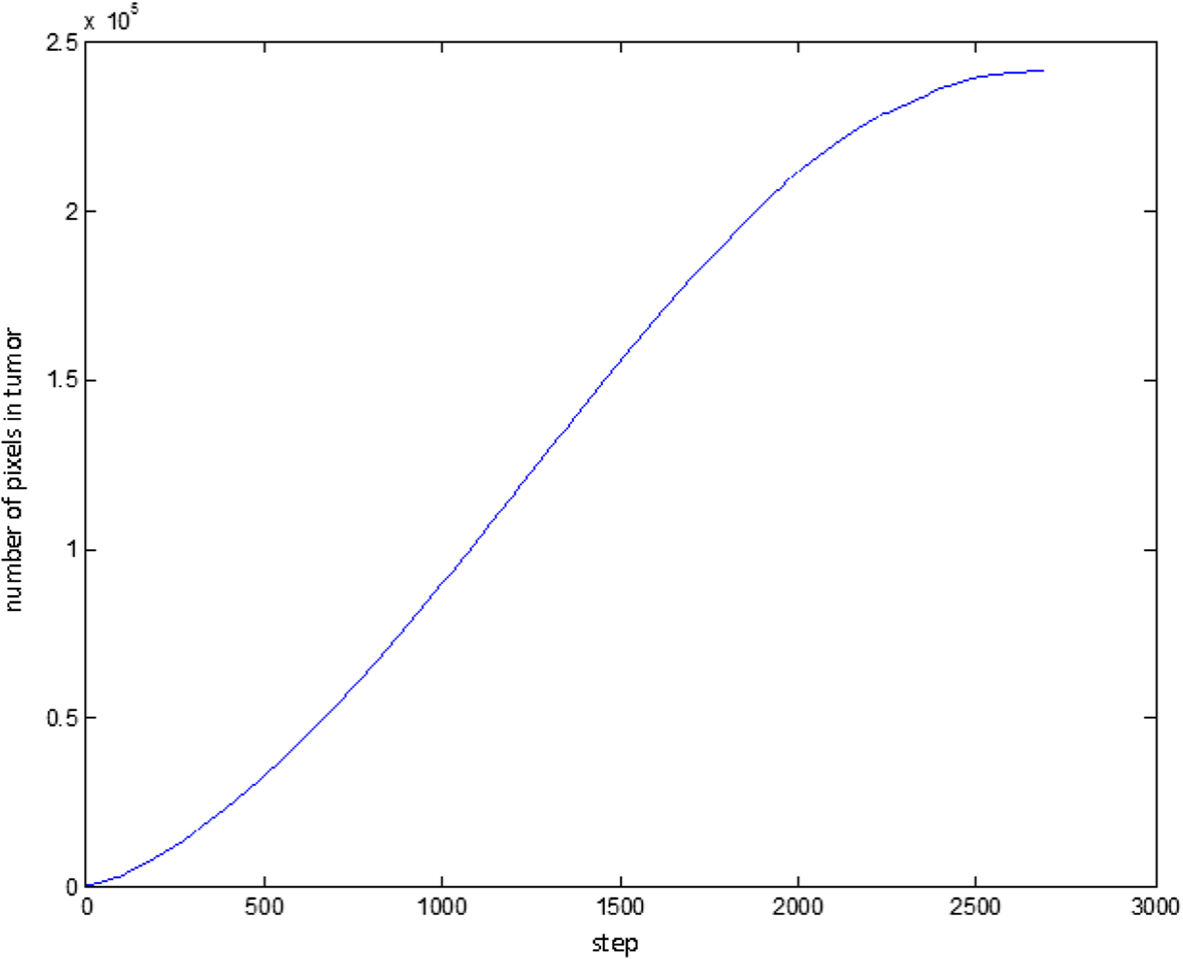
Number of points identified as tumor at different stages of the algorithm

Figure 27 shows the number of pixels that have been identified as tumor points. Obviously, this chart will always be bullish.

## Discussion

Scheming a volume suitable for the maximum flow analysis requires the minimum section related to the maximum flow that can be regarded as an optimal segmentation. A more computationally feasible technique has recently been proposed by Sun [28], while Roy and Cox [29] have established a version of maximum flow examination. In order to efficiently achieve a 3D maximum surface, a two-stage dynamic programming (TSDP) method has been introduced, which allows the computation of a dense disparity map [30]. Because the projected volume works directly in true 3D coordinates, he aimed to output a 3D surface in his suggested voxel volume formulation that represents the entire 3D scene rather than utilizing a disparity map to generate a 2.5D sketch of the scene [31]. Formulating the 3D reconstruction problem as a segmentation issue has some benefits over using the classical dynamic programming method. With segmentation, the optimization is performed along a surface and not along a line. Consequently, it offers the advantage of segmentation procedures to output outlines that wrap back on themselves, while dynamic programming will have trouble following these concave surfaces. Instead of reformulating dynamic programming or similar methods to model concavities and occlusions in 3D reconstruction, the EAG algorithm provides a distinct segmentation by relevant angles from tumor center. Leung [30] has applied a dynamic programming (DP) algorithm to the metric volume to segment the volume without surface evolution calculations, but it observed that a DP examination of the metric volume is meaningless. This is because applying DP indicates a disparity solution, and since a disparity solution is a 2.5D sketch, a multi-valued solution, i.e. the curve winding backwards on itself is not possible. Adversely, the DP algorithm assumes that the volume is Euclidean.

In this study, four dissimilar databases from different labs were used to test each part of the proposed method. In addition, the range of tumors was manually determined in all images (referral websites [22–25]) by an expert radiologist and physician. Finally, the obtained results were compared with the existing results and the acceptance percentage rate were evaluated. The pre-processing was performed in two modes, with and without augmenting contrast of the area growth algorithm, and the results have been presented in Table 4. By raising contrast, the early time of growth improved in all cases.

**Table 4:** The effect of increasing contrast on average decreasing growth time

Database	Initial growth time without increasing contrast (s)	Initial growth time with increasing contrast (s)	Time reduction
DIR	6.07	3.62	40%
LCA	9.59	8.11	15%
NSCLC	4.57	2.90	37%
LIDC	3.01	2.56	15%

The accuracy of “lung area appointing” and “growth area constraint” cannot be confirmed by investigating the acceptance rate. Since these two factors represent an essential reorganization in specimens in which the tumor is affixed to the lung wall, failure to complete these two stages may result in a serious inadequacy in the algorithm for the growth of the area. Table 5 evaluates the rate of acceptance for thresholding default and automatic besides modifying “*comparison quantity*” in each stage. Consequently, modifying *comparison quantity* and auto-thresholding demonstrated an acceptance ratio of a maximum of 12% and 15% increase for LCA and NSCLC databases, correspondingly.

**Table 5:** Acceptance rate (AR) from the effect of thresholding automatic and modifying the *comparison quantity* in each stage

**Database**	**AR without modifying the** *comparison quantity* **in each stage**	**AR with modifying the** *comparison quantity* **in each stage**	**AR with thresholding 20% default**	**AR with thresholding automatic**
DIR	58%	66%	70%	81%
LCA	69%	75%	65%	77%
NSCLC	60%	75%	61%	69%
LIDC	59%	69%	74%	83%

Meanwhile, Table 6 shows the results of the two cases when the growth algorithm starts at one point or several points. As can be seen from these results, the proposed method has represented a significant impact on the acceptance rate.

**Table 6:** The effect of starting the algorithm from one or more points on the accuracy of the results

Database	AR starting from one point	AR starting from several points
DIR	85%	91%
LCA	70%	88%
NSCLC	80%	92%
LIDC	77%	89%

Moreover, Table 7 shows the results of the edge improvement at the end when the edges are corrected or not. As can be seen from these results, the edge correction at the final step has had a great influence on the acceptance rate by a maximum of 13% difference for image LIDC database.

**Table 7:** The effect of edge improvement at the final step on the accuracy of the results

Database	AR without edge improvement	AR with edge improvement
DIR	78%	90%
LCA	85%	93%
NSCLC	79%	89%
LIDC	80%	93%

Finally, an enhanced algorithm covering all preceding procedures was implemented on 60 input images. The results shown in Table 8 revealed that this enhanced algorithm could largely achieve tumor segmentation with sufficient accuracy in a large number of images compared to the primary algorithm. The maximum and minimum AR differences in implementing these algorithms were 13% and 5% for images DIR and LCA, correspondingly.

**Table 8:** AR amounts from implementing primary and enhanced algorithms in tumor area growth (*p*-value < 0.05)

Database	Primary algorithm AR	Enhanced algorithm AR
DIR	78%	90%
LCA	85%	93%
NSCLC	79%	89%
LIDC	80%	93%

The lung density is normally affected by parameters like imaging protocol, physical material characteristics of lung parenchyma, pressure of trans-pulmonary, air volume, and tissue volume. These parameters make it difficult to choose a grey scale segmentation threshold because diverse subjects are probably to need diverse thresholds. Some reports have utilized a single or multiple predetermined thresholds to separate the lungs from the surrounding anatomy [32, 33]. Here in presented algorithm, automatic thresholding was used to select a threshold based on the local characteristics of the color intensity to increase the border recognition. As shown in Table 5, the maximum acceptance rate from the effect of thresholding automatic and modifying the *comparison quantity* in each stage brought about 83% (LIDC) and 75% (LCA and NSCLC), respectively*.* This thresholding is also anticipated to work better for deviations in lung volume where there are major alterations in lung density.

The parameter of Recall [True Positives/(True Positives + False Negatives)] measures the proportion of the positive examples that are correctly identified, while the parameter of Precision [True Positives/(True Positives + False Positives)] evaluates the proportion of the nominated positive examples that are correct. Thus unlike, the false positive rate, it is not dominated by the large number of non-lung boundary pixels. Table 9 summarizes the individual power values measured for different databases.

**Table 9:** Recall and precision parameters in comparison of primary and enhanced algorithms

Database	Primary algorithm		Enhanced algorithm	
	**Recall**	**Precision**	**Recall**	**Precision**
DIR	0.85	0.88	0.9	0.92
LCA	0.83	0.89	0.91	0.93
NSCLC	0.9	0.88	0.92	0.93
LIDC	0.89	0.87	0.91	0.90

Mesanovic et al. [34] have introduced an automatic segmentation algorithm in which the region grows using a hole-filling operation that cannot accurately detect the pulmonary nodule attached to the pleura and ribs. Meanwhile, Bellotti et al. [35] have presented an active contour model in region growing and nodule detection, so that the segmented volume is reduced to about 15% of the original total volume and about 25% of the chest volume. Although their start points were different when selecting the sectional images, the detection rate was 80% by 2.47 false positive results per 15 number of CT scan. In this study with an enhanced algorithm, the minimum acceptance rate for the growth of the tumor area is 88% for DIR database according to Table 8.

In this study, the obtained dice coefficient was near each other for both the primary and enhanced algorithms, respectively, by 0.80 ± 0.02 and 0.92 ± 0.03. It was found that the primary algorithm tends to have greater segmentation variation of 60 image frames with a less error rate in comparison with the pervious study using only four patients CT images [36]. Besides, the p-value of these dice coefficients was less than 0.05 derived from the pairwise Student t-test between two algorithms.

Kalpathy-Cramer et al. [37] have compared the performance of three lung nodule segmentation algorithms via spatial overlap and volume measurements. Their results revealed that the concordance correlation coefficient for algorithmic determination of nodule volume were 0.997 and 0.836, respectively, for repeatability and reproducibility by 95% confidence interval. In addition to that, the mean dice score was 0.95 versus 0.81 (*p* < 0.001 of Wilcoxon rank sum test), correspondingly, and typically was greater for larger nodule volumes. Therefore, this underscores the suggestion to utilize the same software at all-time points in longitudinal researches and when measuring factors such as tumor doubling time.

Jiang et al. [38] have presented a resolution-residual-neural-network method via concatenating features computed simultaneously at multiple image resolutions in dense and incremental ways. Their selected tumors locations were within lung parenchyma, attached to the chest wall, and adjacent to mediastinum in dissimilar sizes. Regardless of tumor size and location, they did not utilize 3D convolutions specially for tumors attached to mediastinum in longitudinal and slice-wise segmentation via ROI-founded training framework. In computing the overlap between the segmentation outcomes and the ground truth, their best performing method had a dice similarity coefficient by 0.75 ± 0.12. Since the tumor in the patients with acquired resistance is difficult to distinguish from the abutting mediastinal pleura at later time points, their algorithm outcomes were not perfect in the over-segmentation process, apart from stopping at a maximum epoch number of 100 in overfitting prevention.

Reamaroon et al. [39] have evaluated an image processing algorithm for lung segmentation in chest radiographs via Total Variation-based Active Contour (TVAC) by 0.86 ± 0.04 average Dice coefficient against 0.74 and 0.64 for random walker and active spline algorithms, respectively. Osareh and Shadgar [40] have presented a segmentation technique based on region aided geometric snake for lung cavities that their model integrated the gradient flow forces with region constraints provided via fuzzy c-means clustering by maximum 0.962 precision. Meanwhile, Kumar et al. [41] have presented a freehand scheming using multi-seed points for selection of ROI along with geometric modeling and implicit surface reconstruction in volumetric nodule extraction. They estimated a discrepancy in their suggested scheme and the manual contouring to be 3.04 ± 1.7 mm and an accuracy of about 70%, but the average accuracy reported in edge detection has been around 57% while the segmentation errors has been occurred near the nodules boundary. Despite the methods used to segment lung nodules, it remains a challenge to reach an acceptable performance limit in user interactions and to adjust several parameters to achieve satisfactory performance.

Here, morphological operators were used to define the lung regions through automatic thresholding along with erosion and dilation operations by removing the background air, then extracting the lung from the thorax area, and finally refining the boundaries of the lung region (*p*-value < 0.05) [42]. Segmentation accuracy was evaluated by calculating the similarity between the segmented region and its corresponding ground truth. Table 10 compares the resulting average accuracy of the suggested algorithms with recently reported algorithms for tumor region expansion and lung nodules detection from CT images.

**Table 10:** Accuracy comparison of the proposed algorithms with the other reports

Refs. & Methods	Accuracy (average)	Size (number of CT scans in dataset)	Database
Leader et al. 2003 [5]	95.8%	101	GE Medical System, Milwaukee
Kumar et al. [41]	70.0%	220	LIDC^a^
Shen et al. 2015 [43]	92.6%	233	LIDC
Wu et al. 2016 [44]	87.6%	60	LIDC-IDRI^b^
Uzelaltinbulat et al. 2017 [45]	97.1%	70	LIDC
Wang et al. 2018 [46]	96.1%	1018	LIDC-IDRI
Xu et al. 2019 [47]	99.1%	2460	various databases
Khehrah et al. 2020 [48]	92.0%	75	LIDC
Javan et al. 2021 [49]	96.0%	1000	Local patients
Primary algorithm (this study)	84.7%	170	DIR-LCA^c^-NSCLC^d^-LIDC
Enhanced algorithm (this study)	92.1%	170	DIR-LCA-NSCLC-LIDC

^a^Lung Image Database Consortium

^b^Image Database Resource Initiative

^c^Lung Cancer Alliance

^d^Non-Small Cell Lung Cancer

Here in this survey, the proposed algorithm via appointing lung area beside automatic thresholding and also starting from several points along with edge improvement may diminish the human errors in interpreting tumor areas and selecting start point of the algorithm by radiologist. The EAG algorithm may be integrated with other methods to precisely align the acceptance percentage. Also, thresholding can be tuned by a color intensity procedure in order to grow the tumor area perfectly. The proposed algorithm has limitations as it can only be applied to a specific area and may not be considered accurately for nodules on the pleura and juxtapleural nodules because the choice of the initial starting point of the algorithm is left to the user and not entirely can be selected automatically. Since the thresholding procedure includes only 20% of adjacent points by default, it is necessary to explore different ranges of the threshold in the next studies. In the proposed algorithm, the size of the tumor is determined visually by the user, which can be done from one starting point or several starting points. Since the diffusion mass transfer equation can describe the tumor growth and disease speed ratio through numerical modeling, the future study will examine this topic. Generally, this issue depends on many factors like age, gender, weight or size, biochemical environments, genetic predisposition and etc. In this study, starting the growth algorithm from multi-point created precise tumor edges. The algorithm also guarantees the independence of the results from the starting point. Future work may address the metastasis tumor area identification by fuzzy interface system and artificial neural network to differentiate between benign and malignant lung nodules.

## Conclusion

The proposed method is independent of whether the image is homogeneous or symmetrical or not, and is also independent of the matrix size, since the image segmentation is first defined by thresholding around the initial tumor and then by assigning points and expanding them. The projected algorithm enhanced tumor detection by more than 18% with a sufficient acceptance ratio of accuracy. Since the enhanced algorithm is independent of matrix size and image thickness, it is very likely that it can be easily applied to other images by first thresholding around any initial contiguous tumor, then assigning points and expanding this through interpolation. Further studies in the future will address the physical and biological phenomena of tumor growth for other images from different imaging modalities.

## Data Availability

All data required to support the results and conclusions of the study have been provided here with the submission.

## Abbreviations

CT: Computed tomography
EAG: Enhanced area growth
LCA: Lung Cancer Alliance
DIR: Deformable Image Registration
AR: Acceptance rate
